# Platinum Nanocatalysts Supported on Defective Hollow Carbon Spheres: Oxygen Reduction Reaction Durability Studies

**DOI:** 10.3389/fchem.2022.839867

**Published:** 2022-02-21

**Authors:** Victor Mashindi, Pumza Mente, Tumelo N. Phaahlamohlaka, Nobuhle Mpofu, Ofentse A. Makgae, Beatriz D. Moreno, Dean H. Barrett, Roy P. Forbes, Pieter B. Levecque, Kenneth I. Ozoemena, Neil J. Coville

**Affiliations:** ^1^ Molecular Sciences Institute, School of Chemistry, University of the Witwatersrand, Johannesburg, South Africa; ^2^ HySA Catalysis Centre of Competence, Department of Chemical Engineering, Catalysis Institute, University of Cape Town, Cape Town, South Africa; ^3^ National Centre for High-resolution Electron-microscopy (nCHREM), Centre for Analysis and Synthesis NanoLund, Lund University, Lund, Sweden; ^4^ Canadian Light Source Inc., Saskatoon, SK, Canada

**Keywords:** platinum, hollow carbon spheres, oxygen reduction (ORR), nanocarbon, pair distribution function, catalysis

## Abstract

The durability and long-term applicability of catalysts are critical parameters for the commercialization and adoption of fuel cells. Even though a few studies have been conducted on hollow carbon spheres (HCSs) as supports for Pt in oxygen reduction reactions (ORR) catalysis, in-depth durability studies have not been conducted thus far. In this study, Pt/HCSs and Pt/nitrogen-doped HCSs (Pt/NHCSs) were prepared using a reflux deposition technique. Small Pt particles were formed with deposition on the outside of the shell and inside the pores of the shell. The new catalysts demonstrated high activity (>380 μA cm^−2^ and 240 mA g^−1^) surpassing the commercial Pt/C by more than 10%. The catalysts demonstrated excellent durability compared to a commercial Pt/C in load cycling, experiencing less than 50% changes in the mass-specific activity (MA) and surface area-specific activity (SA). In stop-start durability cycling, the new materials demonstrated high stability with more than 50% retention of electrochemical active surface areas (ECSAs). The results can be rationalised by the high BET surface areas coupled with an array of meso and micropores that led to Pt confinement. Further, pair distribution function (PDF) analysis of the catalysts confirmed that the nitrogen and oxygen functional groups, as well as the shell curvature/roughness provided defects and nucleation sites for the deposition of the small Pt nanoparticles. The balance between graphitic and diamond-like carbon was critical for the electronic conductivity and to provide strong Pt-support anchoring.

## Introduction

The global dependence on fossil fuels for everyday energy needs is detrimental to the long-term sustainability of the earth. Over the years, scientists have shown that the combustion of fossil fuels for energy is the driver of rising global temperatures, recurring droughts and other adverse weather and climatic conditions (Rashedi et al., 2020). Therefore, research efforts to find new sustainable energy sources and systems that have a neutral carbon footprint with good efficiencies are needed.

The use of hydrogen fuel cells, commonly referred to as proton exchange membrane fuel cells (PEMFCs), could provide one solution to environmental and energy problems. These PEMFCs use platinum or its composites as the cathodes and anodes for the generation of electric currents usable in power machinery and equipment. The critical hydrogen oxidation reaction (HOR, anode) or the oxygen reduction reaction (ORR, cathode) takes place on the Pt (Genorio et al., 2010). Currently, the state-of-the-art PEMFC catalyst is a Pt or a Pt/transition metal bimetallic catalyst placed on a variety of supports. Supports that have been used vary from structured carbons like nanotubes (CNTs), nanofibers (CNFs), carbon black (CB), broken hemispherical hollow carbons (BHCSs) as well as doped metal oxides and carbides (Mao et al., 2019; Jiménez-Morales et al., 2020; Mohideen et al., 2020; Wang et al., 2020; Mashindi et al., 2021). Carbon supports are favoured due to their different structured forms, their lightweight that is important for mobile applications, thermal stability for high-temperature applications, high surface area for nanoparticle deposition and gas diffusion, as well as their earth abundance. In these catalysts, the metal nanoparticles exist in high loadings to mitigate the slow kinetics and high overpotential, especially for ORR at the cathode (Park et al., 2020). These high loadings are one of the drivers of the high cost of fuel cells and their low durability due to agglomeration and dissolution of metal nanoparticles during the stressful events within a fuel cell operation cycle.

The other challenge associated with fuel cells is the modest durability of the carbon supports or the poor conductivity (and low surface area) of other supports like metal oxides. In fuel cells, degradation occurs during load cycling or during the stop-start events where cell potentials can surpass 1.5 V vs. RHE. Above these potentials, carbon is electrochemically oxidized to CO_2_ and Pt is known to dissolve and redeposit on larger clusters facilitating the debilitating processes of dissolution and agglomeration (Sandbeck et al., 2020). These degradation processes lower the surface area of the catalyst materials resulting in lowered activities and efficiency of the fuel cells, especially over time. The long-term goal is thus to develop a highly durable catalyst that is cheap and can sustain operation over 5,000 h as proposed by the United States Department of Energy in its fuel cell blueprint (Ren et al., 2020). Though newer non-carbon supports for Pt have been discovered in the previous decade, the other favorable properties of structured carbons have made it difficult to completely dispose of carbon as the support of choice, hence the continuous study on how a structured carbon can enhance the durability of the fuel cell catalysts (Jackson et al., 2017; Mohamed et al., 2018; Jiménez-Morales et al., 2020). Even though a few studies have been conducted on Pt supported on hollow carbon spheres (HCSs) and nitrogen doped HCSs (NHCSs), detailed studies on durability protocols have not been comprehensively elucidated for these catalyst systems. In one study, Qian and colleagues studied platinum nanoparticles placed on the inner walls of hollow carbon spheres using a dual-templating method. In this method, the Pt was first deposited on silica spherical templates followed by covering with polydopamine and di-block copolymer micelles as soft templates. Subsequently, carbonization, annealing and etching of the silica template resulted in the formation of a Pt@HCS-mesoporous catalyst with outstanding stability in the methanol oxidation reaction. The electrochemical stability was attributed to the porous carbon shells providing pathways and channels for diffusion of reactants but also protecting the inner Pt particles from agglomeration (Qian et al., 2017). Separately, Zhang and co-workers studied Pt nanoparticles with high dispersion supported on hierarchical N-doped porous HCSs for methanol oxidation. The prepared Pt catalysts demonstrated higher methanol oxidation activity and tolerance for CO intermediates compared to commercial Pt/C benchmarks. They ascribed the favorable catalytic properties to the high dispersion of Pt particles due to the presence of the nitrogen species, the porous-thin mesoporous shell and the hollow macroporous core structure of the support (Zhang et al., 2015). Yan and co-workers deposited highly stable small-sized Pt particles on mesoporous hollow carbon spheres for the oxygen reduction reaction. They observed that the supports had a high BET surface area (1,163 m^2^ g^−1^), large pore volume (2.8 cm^3^ g^−1^) and mesoporous structure and they attributed the distribution and dispersion of small-sized Pt particles to these textual properties. In electrocatalytic reactions, they observed that the mass current density on Pt/HCS electrocatalyst was 1.7 times as high as that of commercial Pt/C in ORR. The stability of the electrocatalysts was attributed to the mesopores that provided a physical interaction force between the Pt and the HCSs. The researchers acknowledged both the superiority of Pt nanoparticles in electrocatalysis and the role of the mesoporous hollow carbon spheres. Also in other studies (Yan et al., 2013), the mesoporosity was shown to contribute to pore confinement of metal nanoparticles that enhanced the stability by reducing migration of the metals. However, in all these Pt/HCSs studies, very few comprehensive durability protocols for both the Pt and the carbon support have been reported on the materials (Wang et al., 2006; Wang et al., 2007; Sebastián et al., 2012; Hu et al., 2021).

Therefore, herein we report on the electrochemical activity and load cycling and stop-start durability of Pt nanoparticles supported on HCSs and NHCSs (ca. 40 wt.% loading) and compare the data to a commercial benchmark Pt/C catalyst (Yano et al., 2010; Lu et al., 2013; Lee et al., 2014). The work continues from an earlier paper that investigated placing Pt on broken HCSs (Mashindi et al., 2021). The load cycling and start-stop durability of these materials were investigated and the changes in activity and electrochemical surface area were measured and correlated with the durability data. The new Pt/HCSs and Pt/NHCSs were tested for ORR activity and durability.

Pair distribution functional analysis (combined with other techniques) provided key information on the Pt-C and Pt-NC interactions and allowed for explanation of the data in terms of surface interactions.

## Experimental

Hydrofluoric acid (48%), cetyltrimethylammonium bromide (CTAB), ethylene glycol, absolute ethanol, melamine, methanol (99%), sulfuric acid (98%), platinum acetylacetonate [Pt (acac)_2_, 97%], tetraethyl orthosilicate (TEOS, 98%), resorcinol, formaldehyde (37%), Nafion perfluorinated resin solution (5 wt.% in aliphatic hydrocarbons and water) and ethylene glycol were all purchased from Sigma-Aldrich and used without further purification. Nochromix crystals (Gordax laboratories), ammonia solution (25%, Associated Chemical Enterprises), absolute ethanol (99.6%, MK chemicals), perchloric acid (70%, Suprapur, Merck), ultrapure water (18.2 MΩ cm, Merck-Millipore), alumina polish (0.05 and 0.1 µm and polishing cloths, Buehler), argon gas (99.99%, Afrox), oxygen gas (99.99%, Afrox), nitrogen gas (99.99%, Afrox), were obtained and used without further purification.

### Synthesis of the Silica Templates, HCSs, NHCSs, Pt/HCSs and Pt/NHCSs Materials

The method described by Stober and colleagues was used, with minor changes, to prepare spherical silica templates (Stöber et al., 1968). In the synthesis method, absolute ethanol (490 ml) was stirred together with tetraethyl orthosilicate (TEOS, 50 ml), deionized water (50 ml) and ammonia solution (30 ml, 25%) at room temperature for 6 h. The silica particles were separated from the solution by centrifugation at 18,000 rpm and washed with 300 ml of a 50:50 vol.% absolute ethanol:deionized water solution. The silica particles were dried in an oven at 100°C overnight followed by calcination at 500°C. The formed silica particles (4.8 g) amounted to a yield of 92% based on the TEOS used.

To form the HCSs, a resin of resorcinol-formaldehyde (RF) was deposited on the silica (SiO_2_) template. In the procedure, the silica powder (1.5 g) was dispersed by sonication in a solution of absolute ethanol (105 ml) and deionized water (25 ml). The surfactant and porogen, CTAB (2 g), was added to a premixed solution of 37% formaldehyde solution (0.3 ml) and resorcinol (0.3 g) and 25% NH_4_OH solution (3 ml). The procedure proceeded with magnetic stirring for 24 h leading to the formation of SiO_2_@RF as the solution turned deep brown with vigorous stirring. After 24 h the products were filtered and washed with 500 ml of deionized water, followed by drying in an oven overnight at 100°C (Mente et al., 2021).

The dried brown product (SiO_2_@RF) was transformed into a carbonaceous material using a tubular horizontal furnace saturated with argon at 900°C. Typically, the SiO_2_@RF (50 mg) was loaded into a quartz boat and placed in the centre of the furnace, under Ar flowing at 50 ml min^−1^. The furnace was ramped up to 900°C at a heating rate of 10°C min^−1^ and kept isothermal for 2 h. The quartz boat was cooled using a fast flow of compressed air for 30 min. The black soot (SiO_2_@C) was etched using a 10% HF solution in water (100 ml) for 24 h to remove the SiO_2_ template. To prevent the room temperature evaporation of the HF solution, the etching was conducted in a sealed Teflon container placed in a fume hood. The solution was vacuum filtered, washed with copious amounts of DI water and dried in an oven at 100°C overnight. The formed HCSs were annealed in a tubular horizontal furnace at 900°C for 2 h at a heating rate of 10°C min^−1^ under an argon flow rate of 50 ml min^−1^. The formed HCSs (600 mg) gave a yield of 88% based on the starting resorcinol and formaldehyde used.

The NHCSs were prepared from SiO_2_@RF and melamine using the same tubular horizontal furnace. Typically, 0.2 g of SiO_2_@RF was mixed with 0.2 g of melamine in a glass vial containing methanol (5 ml) followed by sonication for 30 min. The products were dried in an oven at 70°C for 2 h. The SiO_2_@RF-melamine was transformed to SiO_2_@NHCSs using the same procedure used for the SiO_2_@HCSs, after etching with HF to give NHCSs (Dlamini et al., 2020).

The nominal 40 wt.% Pt/HCSs or Pt/NHCSs catalysts were prepared by dispersing HCSs or NHCSs (20 mg) and Pt (acac)_2_ (27 mg) in a solution of absolute ethanol (100 ml), deionized water (10 ml) and ethylene glycol (10 ml) and the mixture sonicated for 30 min to allow for thorough mixing. The composite, placed in a round bottom flask (attached to a reflux condenser), was then placed in an oil bath heater. The bath was heated to 200°C at a slow heating rate of 2.5°C min^−1^ and kept isothermal for 2 h while the reaction continued under reflux. The reactor was allowed to cool naturally to room temperature, and the products were filtered under vacuum and washed twice with 300 ml deionized water followed by drying at 100°C (Teranishi et al., 1999).

### Catalyst Characterization

TEM analysis was performed using a Tecnai T12 transmission electron microscope operating at 120 kV. Sample preparation was done by dispersing (under sonication) ca. 10 mg of each catalyst in 1 ml ethanol for 5 min. About 3 drops of the material rich ink were deposited onto a lacey carbon copper grid, and analysis by TEM was done after drying the samples in air. Particle size distributions of the Pt particles, HCSs and NHCSs, were obtained by measuring at least 100 particles using ImageJ. The powder X-ray diffraction measurements were carried out on a Bruker D2 phaser diffractometer with a Cu Kα radiation source operating at 40 kV to determine the crystalline phases present in the catalyst with 2ϴ between 10° and 90°. Indexing the compounds detected by the PXRD technique was achieved using the EVA software. Particle sizes of platinum particles were calculated using the EVA software embedded Scherrer equation Thermal stability and metal loading of the catalysts was performed with a Perkin-Elmer STA6000 (TGA) analyser using N_2_ as the purge gas (20 ml min^−1^) and air for combustion (10 ml min^−1^) and a heating rate of 10°C min^−1^. Nitrogen adsorption/desorption was measured using a Micromeritics Tristar 3000 surface area and porosity analyser set at −195°C, with sample degassing conducted at 150°C overnight. Raman spectroscopy measurements were performed on a Horiba Jobin-Yvon Raman spectrometer with a laser wavelength of ( λ = 514 nm). The XPS measurements were carried out using a Thermo Scientific ESCALAB 250Xi spectrometer with a monochromatic Al Kα (1,486.7 eV) source operating with an X-ray power of 300 W. Total scattering data were collected on the Brockhouse high-energy wiggler beamline at the Canadian Light Source using a wavelength of *λ* = 0.2081 Å and a PerkinElmer XRD1621 area detector placed 160 mm after the sample. The data were processed using GSAS-II (Toby and Von Dreele, 2013). The Qmax used to produce the PDF of the measured samples was 23.4 Å^−1^. The instrumental resolution parameters Qdamp and Qbroad, as defined in PDFgui software, were determined by fitting a Ni powder standard measurement. Further data analysis was done using the xPDFsuite.

### Electrochemical Characterization

The electrochemical experiments were performed in a three-electrode cell at room temperature (approx. 25°C) in a solution of 0.1 M HClO_4_ purged with nitrogen for cyclic voltammetry (CV) or oxygen for the oxygen reduction reaction (ORR) experiments. The counter electrode was a high surface area Pt wire and the reference electrode was Ag/AgCl (3.0 M KCl). All Ag/AgCl potentials were converted to RHE by calibrating the potential between the reference electrode and an *in-situ* RHE prepared by saturating a clean Pt wire immersed 0.1 M HClO_4_ with hydrogen gas. Arbitrarily, no *i*R drop correction was conducted. For Pt and support durability studies, the high surface area Pt wire was replaced with a high surface area gold counter electrode. A catalyst thin film coated glassy carbon (GCE) electrode with a working area of 0.196 cm^2^ was used as the working electrode (WE). The catalyst inks were prepared by dispersing about 5 mg of the catalyst in a solution of ultrapure water (1.5 ml, 18.2 MΩcm), isopropyl alcohol (3.5 ml, HPLC grade) and Nafion perfluorinated resin solution (20 µL, 5 wt. % in a mixture of lower aliphatic alcohols and water) followed by a low-temperature 30 min sonication. The electrochemical cell, electrolyte volumetric flasks, purge tubes and reference electrode bridge tubes were all thoroughly cleaned with a solution of Nochromix and concentrated sulfuric acid followed by multiple rinses in Merck-millipore ultra pure water. A Biologic SP300/VMP300 potentiostat coupled to a Gamry RDE (rotating disk electrode) 710 Rotator was used for CV and RDE measurements.

The potential of the WE was cycled between 0.0 and 1.2 V vs. RHE for 100 cycles at 100 mVs^−1^ until a reproducible CV was obtained. The scan rate was then reduced to 50 mVs^−1^ and the third cycle was used for the calculation of the electrochemical active surface area (ECSA) assuming a monolayer charge associated with hydrogen adsorption of 210 µCcm^−2^. The area under the CV curve, representing the charge associated with the underpotential deposition of hydrogen (Q_DES_), was integrated and used for the calculation of ECSA according to the equation:

$$\mathrm{ECSA=}\left(Q_{\mathrm{DES}}\right)/\left(\mathrm{210}\mu \mathrm{C c}m^{\mathrm{-2}}\times L_{\mathrm{Pt}}\right)$$ (1)

The L_Pt_ is the loaded amount of Pt particles on the 0.196 cm^2^ surface area of the working electrode. Oxygen reduction reaction (ORR) I (current)-V (voltage) polarization curves were obtained at 1,600 rpm on the electro-catalyst coated working electrode. The WE was cycled at 10 mV s^-1^ between 0.0–1.2 V vs. RHE in the cathodic direction. To correct for non-ORR background current, the LSV obtained in the nitrogen saturated electrolyte without rotation was subtracted from that obtained from the oxygen saturated electrolyte. The kinetic ORR currents (I_k_) were extracted from the measured ORR currents (I) and the limiting currents (I_lim_) determined at 0.4 V vs. RHE using the Koutecky-Levich equation.

Finally, kinetic currents were normalised with the ECSA and the initial Pt mass loading to obtain the surface area-specific activity (SA), Eq. 2, and the mass-specific activity (MA), Eq. 3, respectively (Garsany et al., 2010).

$$\mathrm{SA=}I_{k}/\left(Q_{\mathrm{DES}}/\mathrm{210}\mu \mathrm{C c}m^{\mathrm{-2}}\right)$$ (2)

$$\mathrm{MA=}I_{k}\left(\mu \mathrm{A c}m^{\mathrm{-2}}\right)/L_{\mathrm{Pt}}\left(\mu \mathrm{g c}m^{\mathrm{-2}}\right)$$ (3)

Durability load cycling was carried out after the measurement of the beginning of life (BOL) CV and ORR activity. Initially, the WE was cycled between 0.0–1.2 V vs. RHE at 100 mV s^−1^ to clean the catalysts of any impurities and contaminants and to produce a reproducible CV. Load cycling for catalyst durability measurements was carried out in a 0.1 M HClO_4_ solution at 25°C, in a nitrogen saturated electrolyte. The WE was cycled at 50 mV s^−1^ between 0.6–1.0 V vs. RHE. Load cycling was carried out in units of 10, 100, and 1,000 cycles until when the 6,000th cycle was reached after 27 h of continuous cycling. As Pt durability was under investigation, in all durability experiments, the Pt counter electrode was replaced with a gold counter electrode of high surface area (Inaba, 2009).

Start-stop durability cycling was carried out after obtaining the beginning of life (BOL) ECSAs of the three catalysts. The WE electrode was cycled between 1.0–1.6 V vs. RHE at a scan rate of 50 mV s^−1^ in an N_2_ saturated 0.1 M HClO_4_ electrolyte at 25°C. Cycling was carried out for a total of 6,000 cycles with ECSA CVs sampled after 10, 100 and 1,000 cycles until the 6,000th cycle was reached after 27 h of continuous cycling and argon saturation (Ohma et al., 2011).

## Results and Discussion

### Physicochemical Properties

The Raman spectra of the HCSs and NHCSs were recorded to determine the extent of carbon graphitization and the presence of defects in the material. The Raman spectra (Supplementary Figures SI1A,B) were deconvoluted into their respective D, D1, D2 and G bands (Ferrari and Robertson, 2004). The G band is attributed to the Raman vibration of sp^2^ hybridized carbons while the D band is attributed to the Raman vibration of sp^3^ hybridized carbons. The extent of graphitization was measured by the ratio of the D and the G band areas (I_D_/I_G_ ratio). As expected, the HCSs with an I_D_/I_G_ ratio of 0.98 were more graphitic when compared to the NHCSs, with an I_D_/I_G_ ratio of 1.22. The nitrogen groups in the NHCSs introduced carbon vacancies in the structure of the NHCSs resulting in defects from displacement of carbon atoms by the nitrogen (Ewels and Glerup, 2005).

**FIGURE 1:**
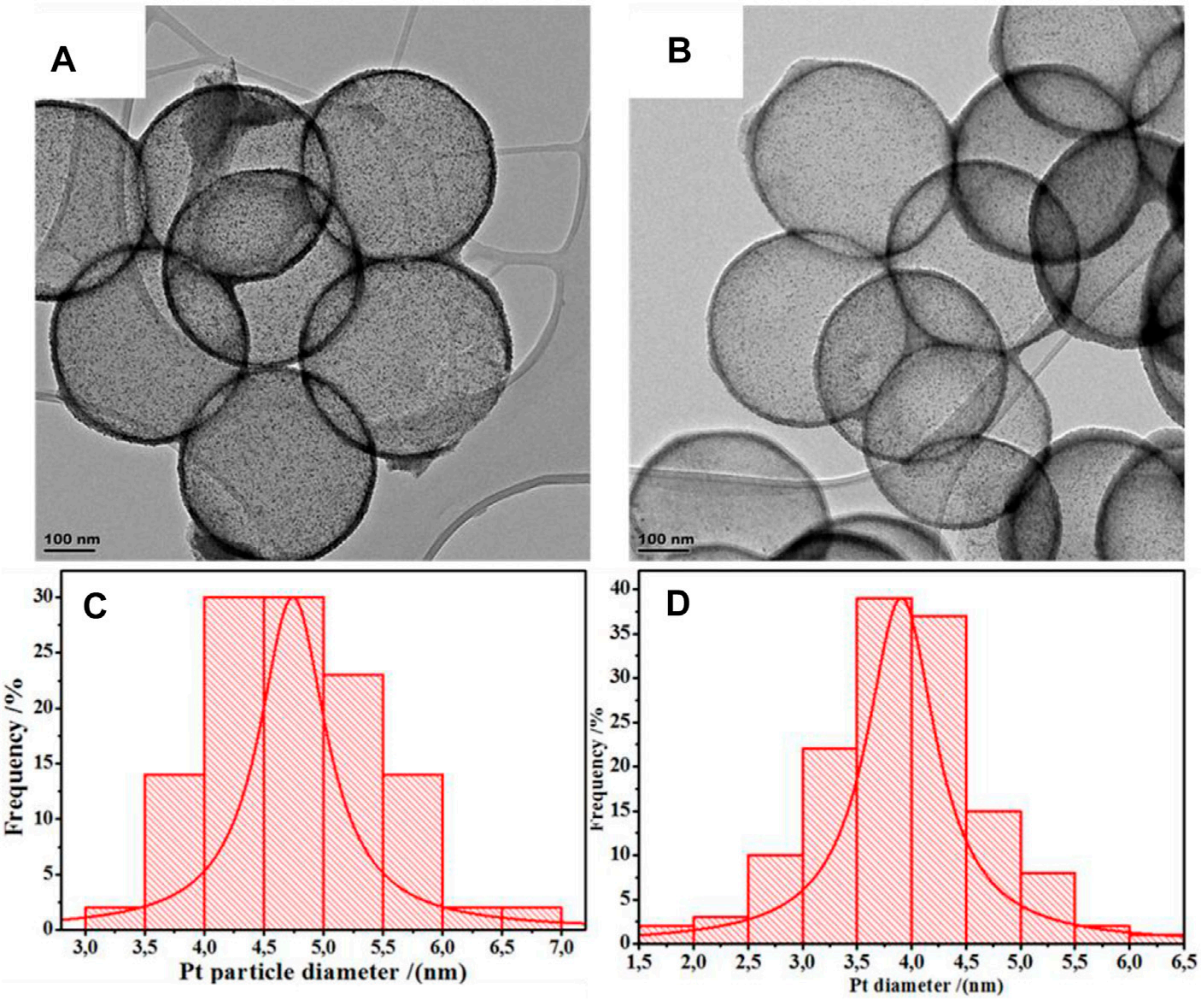
TEM image of **(A)** Pt/HCSs, **(B)** Pt/NHCSs, and particle size distribution of Pt on Pt/HCSs **(C)**, and particle size distribution of Pt on Pt/NHCSs **(D)**.

TEM images indicated the formation of Pt nanoparticles with a spherical morphology (Figure 1, Supplementary Figure SI1), with measured particle sizes of 3.9 ± 0.5 nm and 3.8 ± 0.6 nm for the Pt/HCSs and Pt/NHCSs respectively.

A high degree of Pt dispersion and lack of agglomeration is suggested by the small inter-particle distances of the Pt nanoparticles deposited on both supports (6.5 ± 1.9 nm for Pt/HCSs and 6.1 ± 1.1 nm for Pt/NHCSs (Figure 1, Supplementary Figure SI1). The presence of defects and electron-rich nitrogen functionalities on the supports provides numerous nucleation sites for Pt clusters during deposition, resulting in small Pt-Pt cluster distances (Galeano et al., 2014). It is suggested that during Pt deposition, the charge is transferred from the Pt metal particles to the π –conjugated system of the aromatic rings in the carbon structure as well as to the nitrogen groups supported on these carbon structures due to differences in electronegativity. This increase in electron density and uniformity on the support surface then promotes the deposition of particles of smaller size with small Pt –Pt cluster distances (Palaniselvam et al., 2014). Also, the N doped carbon distorts the surface of the supports causing distorted atomic layers on the surface and some dangling bonds which acts as the capture sites for fixing the Pt nanoparticles on the walls of the HCS/NHCSs (Du et al., 2008).

The HCSs and NHCSs were analysed using TGA (Supplementary Figures SI1C,D) to investigate the thermal stability of the materials under oxidizing conditions. Both the HCSs showed the expected thermal stability (HCSs at 660°C; NHCSs at 658°C). The TGA and DTGA profiles of Pt/HCSs and Pt/NHCSs recorded the residual PtO/PtO_2_ species with Pt content >35 wt.% similar to the nominal 40 wt.% of both the Pt/HCSs and Pt/NHCSs catalysts. The deposition of Pt nanoparticles enhanced the combustion of the carbon, through the expected catalytic reaction of carbon with Pt (Mashindi et al., 2021) and consequently lowered the thermal stability of the materials as shown by the lower decomposition temperatures of the Pt/HCSs The decomposition of Pt/NHCSs also occurred at a lower temperature compared to the NHCSs, in agreement with the presence of defects on the NHCSs, as shown by Raman spectroscopy.

The surface area and porosity of the materials were measured using the BET technique. The materials demonstrated a type IV BET isotherm and microporosity (Supplementary Figure SI3). The pristine HCSs and the NHCSs had high BET surface areas of 832 and 604 m^2^g^−1^, respectively. The observed reduction in BET surface area and porosity after deposition in the Pt/HCSs and Pt/NHCSs respectively (Supplementary Table SI1) was attributed to pore blockage by the metal nanoparticles. The pore blockage is caused by both the “top of pore” and the “in pore” deposition of Pt, with the latter resulting in the confinement of the Pt particles (Galeano et al., 2012). The pore confinement in these supports was confirmed by the dark-field STEM images and elemental maps of Pt particles, which appear to be highly concentrated in the shell of the supports (Supplementary Figure SI4). Also, in these shell regions of the supports, the Pt maps indicate more than a single layer of Pt particles, indicating penetration into the HCSs/NHCSs shells by the Pt particles as it occupies the pores of the support. This is in agreement with studies on broken hemispherical carbon that were similarly prepared (Mashindi et al., 2021).

**FIGURE 2:**
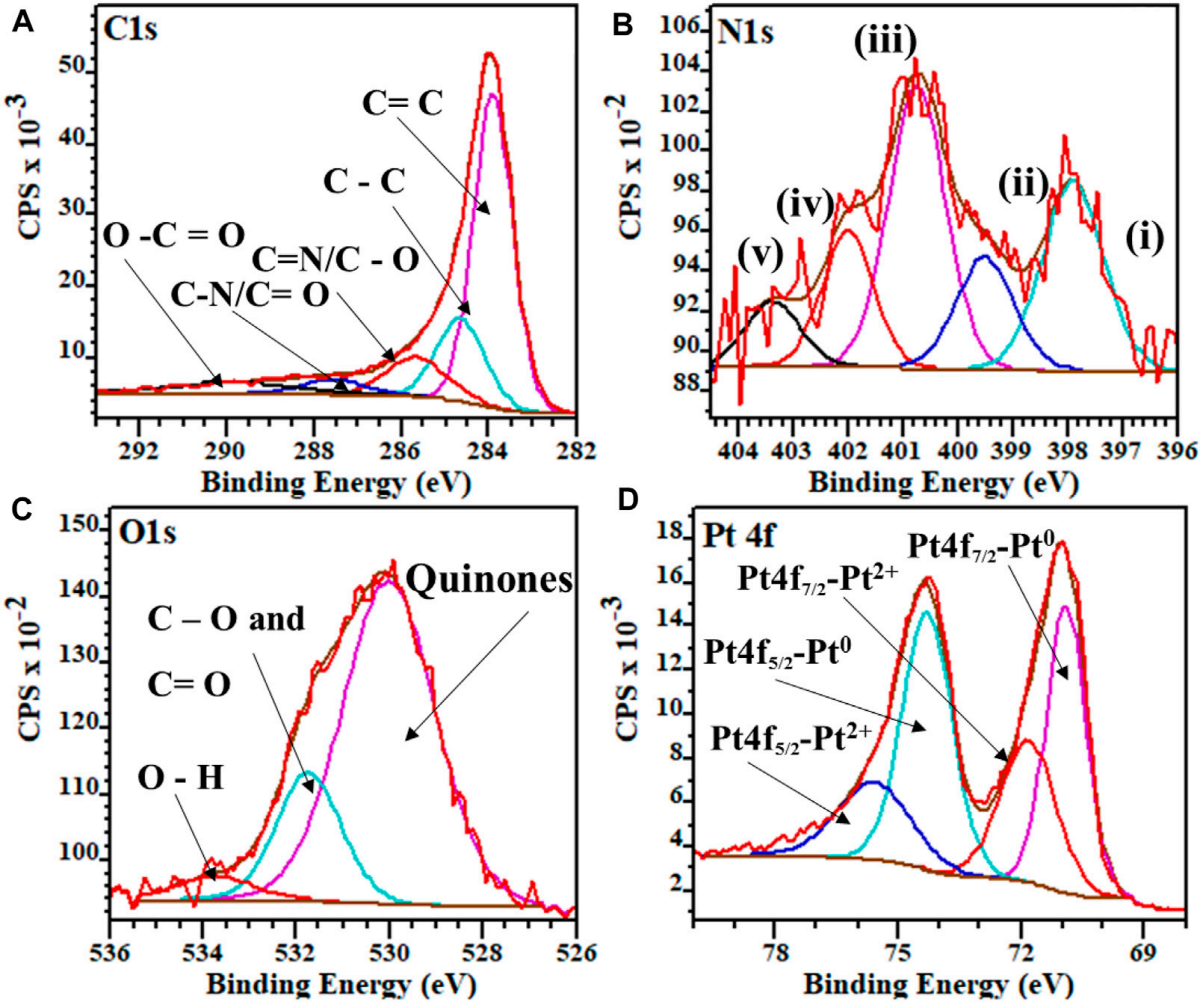
Deconvoluted high-resolution XPS spectra for Pt/NHCSs **(A)** C1s, **(B)** N1s **(C)** O1s and **(D)** Pt 4f.

The XPS survey spectra for both Pt/HCSs and Pt/NHCSs are given in Supplementary Figure SI5 and the deconvoluted spectra for Pt/NHCSs in Figure 2. The C1s spectra we deconvoluted (Figure 2A) into the respective sp^2^ hybridized carbon, C=C (284.0 eV), amorphous diamond like carbon with sp^3^ hybridization, C–C (284.6 eV), C=N/C-O (285.8 eV), C–N/C=O (287.6 eV) and finally O–C=O (289.9 eV). The C=N and C-N bonds indicate the successful incorporation of the nitrogen functionalities into the carbon matrix of the NHCSs (Matsoso et al., 2016). The N1s spectra of the Pt/NHCSs was deconvoluted (Figure 2B) and revealed peaks for pyridinic N (i, 398.3 eV), pyrollic N (iii, 400.7 eV), graphitic N (iv, 401.9 eV), oxidized N (v, 403.1 eV) and metal bonded N (metal-N-pyridyl) nitrogen (ii, 399.7 eV) groups (Chen et al., 2017). The total contribution of the various N species was pyridinic (i, 30%), pyrollic N (iii, 40%), graphitic N (iv, 12%), oxidized N (v, 8%) and the metal bonded N (ii, 10%). The N content of Pt/NHCSs was found to be 7.4%. Deconvolution of the O1s spectra of the Pt/NHCSs (Figure 2C and Supplementary Figure SI5) showed the presence of quinones (530.1 eV), C=O and C–O bonds (531.8 eV), and terminal O–H bonds (533.9 eV) (Melke et al., 2016). The deconvoluted Pt 4f spectra (Figure 2D), indicate the presence of mostly metallic Pt and smaller amounts of oxidised Pt particles. The Pt 4f spectra were deconvoluted into the zero oxidation state metallic Pt, Pt4f_7/2_ (71.1 eV) and Pt4f_5/2_ (74.9 eV) and the +2 oxidation state of the Pt, Pt4f_7/2_ (72.1 eV) and Pt4f_5/2_ (76.2 eV), respectively, (Lu et al., 2016).

The crystallinity of the materials was investigated using PXRD. Both the HCSs and NHCSs showed a structure typical of a mix of both graphitic and amorphous carbons, characterized by the broad 2ɵ PXRD peaks (25 and 45°) (Mente et al., 2021). The Pt on the HCSs and NHCSs showed reflections characteristic of the fcc unit cell of Pt (Sravani et al., 2020) (Supplementary Figure SI6). Using the Scherrer equation, the Pt crystallite sizes were determined to be 4.5 ± 0.8 and 4.3 ± 0.8 nm for Pt/HCSs and Pt/NHCSs, respectively.

**FIGURE 3:**
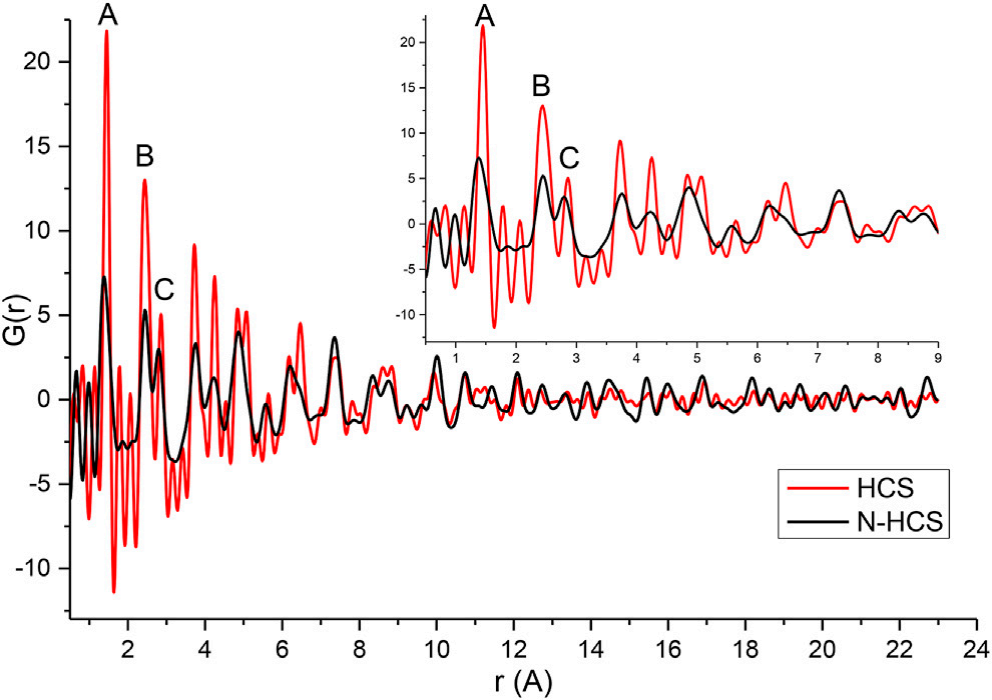
Experimental G(r) of HCSs and NHCSs.

To further explore the effect of nitrogen on the HCSs structure, total scattering data were obtained and are plotted in Figure 3, showing the experimental G(r) of the HCSs in red, and the NHCSs in black. Considering the pristine HCSs first, the peak position values denoted by A, B, and C closely match the expected real space values for in-plane carbon-carbon bond distances in the aromatic-type ring of graphite/graphene for the first three coordination spheres (Mildner and Carpenter, 1982; Krzton and Niewiara, 1995). The first peak (A) in G(r), at 1.45 Å, corresponds to the C-C bonds with three nearest neighbours to carbons with sp^2^ bonding. The second peak (B) at 2.44 Å represents the distance between the three atoms coordinating a central carbon or the shortest diagonal in the hexagon. The third peak (C) at 2.86 Å, which is twice the first C-C distance, is the second, long diagonal in the hexagon. As can be seen from the simulation of graphite C-C bond distances in Supplementary Figure SI7. PDF data can easily distinguish between different carbon coordination environments.

The first peak, A, in the HCS is slightly shifted to a higher r with an integrated peak area of 3.24 implying the support also contains sp^3^ bonds originating from a R-3m rhombohedral diamond-like structures, albeit in lesser amounts relative to the graphite/ene phase (Dmowski et al., 2012). Peak A in the nitrogen doped HCSs deviates from the expected *r* values due to the presence of pyridinic, pyrrolic, and graphitic nitrogen within the structure characterised by a peak position at 1.36 Å, in addition to peak broadening. Pyridinic type N defects are characterised by a peak at 1.33 Å due to the shortening of the C-N bond in comparison to the C-C bonds at 1.45 Å. As the NHCSs consist of a convolution of these bonds, a shortening and broadening of the bond peak position occurs. Further, a reduction in the diagonal length across the hexagon is observed from the 2.86 Å to 2.77 Å due to nitrogen substitution in the structure.

The carbon plane for both samples shows buckling as the position of the third coordination sphere (C) is not exactly half that of the second coordination sphere (B) (Dmowski et al., 2012). The NHCSs show increased buckling of the carbon plane compared to the pristine sample due to nitrogen doping. Furthermore, broadening of the NHCSs third coordination sphere (C) shows greater variations in the bond distances resulting from the strain induced by the buckling of the carbon plane. The broadening is seen in peaks A, B, and C and is a feature arising from highly defective structures in the NHCS. Broadening is also noted at real space distances coinciding with the peaks at 4.23, 4.86, 5.57, and 6.20 Å.

A gradual reduction in structural coherence with increasing distance (increasing r) from the scatterer is noted along the graphene sheet. This is likely due to the curvature of the sheets to form spheres, and the distribution of varying degrees of curvature in the sample, rather than from termination of sheet fragments. The effective atomic density of the scattering volume can be estimated from the slope of the measured G(r). For the HCSs and NHCSs samples the atomic density is 0.095 and 0.105 atoms/A^3^ respectively which is just below the atomic density of graphite at 0.11 atoms/A^3^ (Dmowski et al., 2012). No interlayer graphitic peaks are present in the PDFs (Supplementary Figure SI8). All peaks can be accounted for with the graphene, however, there is significant turbostratic and positional disorder from one graphene sheet to the next and the sheets are not stacked in perfect unison. This is probably a natural consequence of the curvature of the sheets when forming spheres and creating general disorder (Kane et al., 1996).

Atomic correlations are more significantly damped beyond 13 Å for HCS than NHCS. This is the range corresponding to the lateral coherence of the structures and also where stacking differences are important. This behaviour of G(r) indicates that short-range two-dimensional atomic order is similar to graphite but stacking along the c-axis is strongly disordered (Petkov et al., 1999). However, this is expected due to the relaxation of the edge atoms or strain induced by curvature. With increased deformation, tilting or folding of the layers to form spheres combined with weak van der Waals bonds between the layers result in structures that are prone to turbostratic disorder (Ramos-Sanchez and Balbuena, 2013).

**FIGURE 4:**
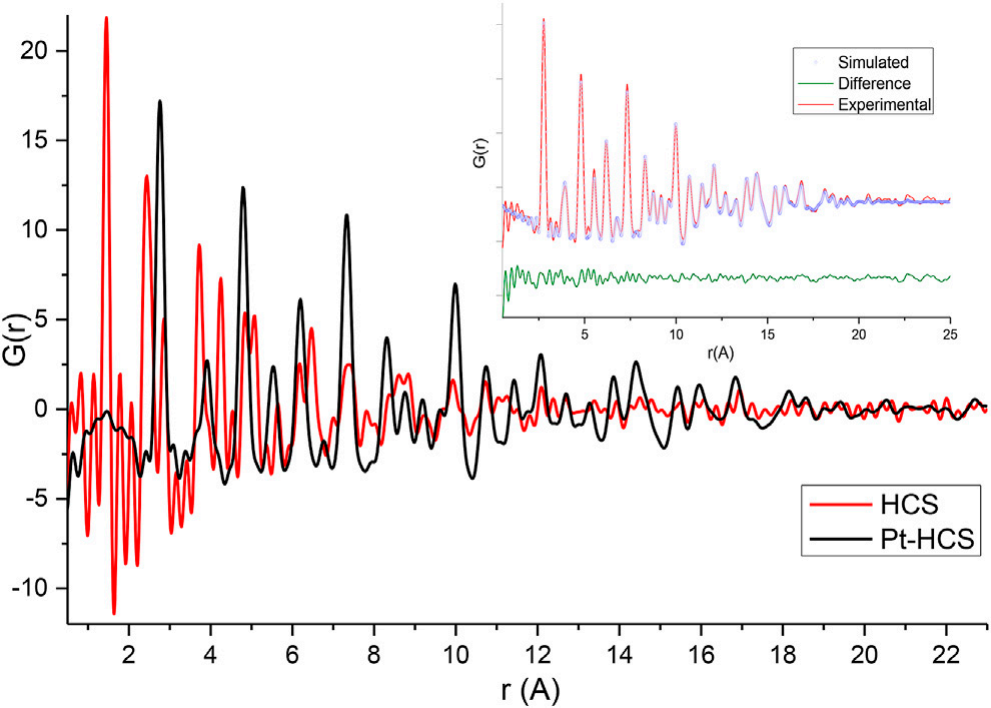
Experimental G(r) of HCSs and Pt/HCSs. Insert—shows the PDF fitting of Pt/HCSs.

**FIGURE 5:**
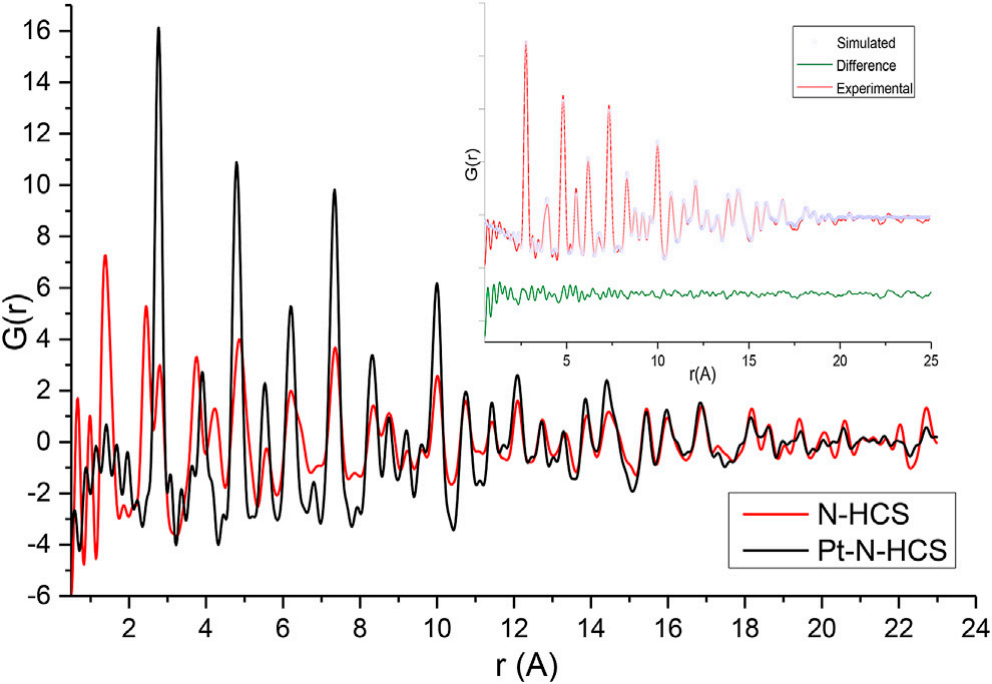
Experimental G(r) of NHCSs and Pt/NHCSs. Insert—PDF fitting of Pt/NHCSs.

The effect of the addition of Pt to the HCSs and N/HCSs was also evaluated from total scattering data. The PDFs of HCSs and Pt/HCSs are shown in Figure 4, and the PDFs of NHCSs and Pt/NHCSs are shown in Figure 5. The high Pt loading of 40 wt.% coupled with the large scattering cross section of Pt results in a Pt dominated PDF signal.

Fitting of the PDFs revealed mean Pt particle sizes of 2.6 and 2.4 nm for the Pt/HCSs and Pt/NHCSs, respectively. It is important to contrast the Pt particle sizes determined from XRD, TEM and PDF measurements. Considering XRD, Bragg diffraction has limitations in accurately determining particle sizes below 5 nm while the resolution and magnification of the TEM images likely result in an underrepresentation of very small particles smaller than 2.5 nm. PDF is highly sensitive to small particles as both Bragg and diffuse scattering are detected resulting in a more accurate particle size determination. The interaction of the Pt particles with the carbon surface has been shown to result in a local rearrangement of carbon atoms, with the stronger the adsorption energy, the larger the C–C bond elongation (Ramos-Sanchez and Balbuena, 2013). As seen in the TEM results, a distribution of Pt particle sizes is present on the surface of the spheres with high dispersion resulting in unequal Pt-carbon interactions that in turn result in variations in the bond lengths which further increases the disorder and defects in the HCS and NHCS structures.

The interaction energy between Pt and graphitic carbon has contributions from orbital hybridisation and van der Waals interactions. The van der Waals interactions are as large as the covalent bond contribution and cannot be neglected (Ramos-Sanchez and Balbuena, 2013). The van der Waals forces are needed to describe the system and contribute to maintaining the Pt linked to the carbon surface. Even though the adsorption energy of Pt has been shown to be weak *via* DFT calculations, this adsorption energy is proportional to the Pt particle sizes and has been shown to have profound effects on the Pt cluster properties (Ramos-Sanchez and Balbuena, 2013).

The lack of well-defined first, second, and third neighbour C-C distances clearly indicates that the aromatic-type rings are heavily distorted when Pt is present pointing to a significant rearrangement of the support structure caused by Pt addition. It must be noted that the carbon signal is also significantly dampened due to the presence of Pt at high wt% loadings (Li and Lannin, 1990). It is not surprising that no well-defined sheet-like patterns are observed in the corresponding TEM images of these samples.

### Catalyst Activity

**FIGURE 6:**
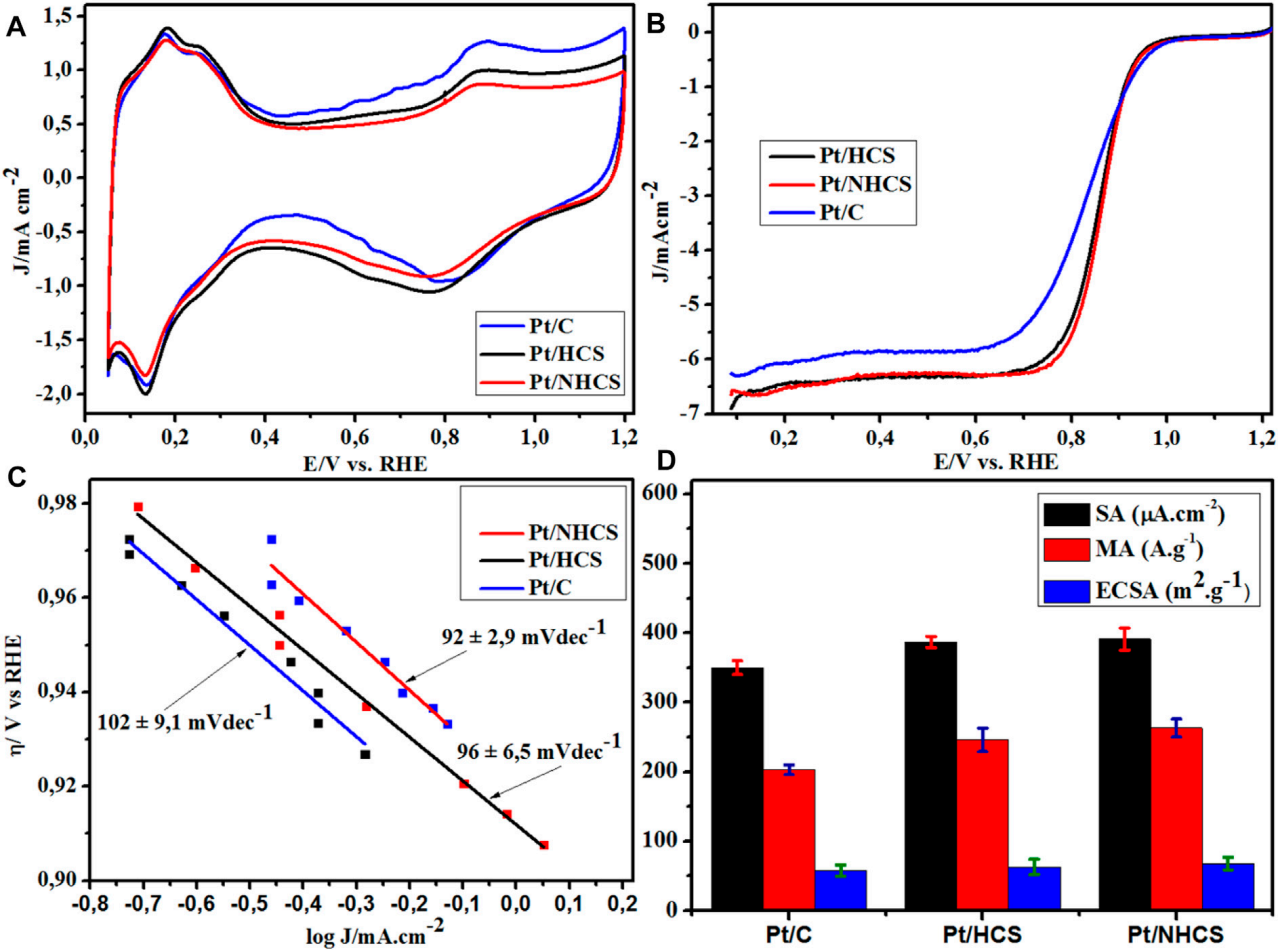
The **(A)** cyclic voltammograms, **(B)** ORR polarization I-V curves, **(C)** Tafel plots and **(D)** ORR activity and ECSA data for the Pt/HCSs, Pt/NHCSs and the commercial benchmark Pt/C.

The Pt materials were studied using cyclic and linear sweep voltammetry for electrochemical surface area (ECSA) and ORR activity. The materials demonstrated a standard Pt/C cyclic voltammogram (Figure 6A), with typical peak features for hydrogen desorption, oxidation of the Pt, reduction of the Pt and adsorption/desorption of hydrogen (Garsany et al., 2010). The activity trends of the materials were obtained from the measured linear sweep voltammograms (LSVs), (Figure 6B). The Pt/NHCSs showed an earlier onset, higher half-wave potential (E_1/2_) and kinetic current than the Pt/HCSs. The least active was the commercial benchmark Pt/C catalyst. The ORR onset, ECSAs, kinetic current and E_1/2_ data are summarized in Table 1. The LSV ORR data was modelled to the Tafel equation,

η=a+b⁡log(j) (4)

where η is the ORR overpotential, a is the Tafel constant or the over potential intercept of the Tafel plot, b is the Tafel slope and (j) is the ORR current density at a specific overpotential. The Tafel plots for the Pt/HCS, Pt/NHCS and Pt/C are shown in Figure 6C. The Pt/NHCSs has the lowest Tafel slope of 92 ± 2.9 mVdec^−1^ compared to 96 ± 6.5 mVdec^−1^ (Pt/HCSs) and 102 ± mVdec^−1^ (commercial Pt/C), thereby confirming that ORR kinetics are more favourable on the nitrogen-doped catalyst, showing superiority to the commercial benchmark Pt/C (Chen et al., 2020).

**TABLE 1:** Calculated ECSA and ORR activity for the Pt/HCSs, Pt/NHCSs and the commercial benchmark Pt/C catalysts. MA, SA, I_k_ measurements obtained at 0.90 V vs. RHE.

Catalyst	ECSA/(m^2^g^−1^)	MA (0.90 V)/(Ag^−1^)	SA (0.90 V)/(µA cm^−2^)	I_k_ (0.90 V)/(mA cm^−2^)	E_1/2_ V vs. RHE	E _onset_ V vs. RHE
Pt/C	58 ± 8	203 ± 7	350 ± 10	2.7 ± 0.6	0.831	1.022
Pt/HCSs	63 ± 11	246 ± 17	387 ± 8	2.9 ± 0.8	0.854	1.027
Pt/NHCSs	68 ± 9	263 ± 13	391 ± 16	3.1 ± 0.2	0.862	1.028

**TABLE 2:** Comparison of the activity of the Pt/HCSs, Pt/NHCSs and commercial Pt/C before and after 6,000 durability cycles.

Catalyst	MA/(0.9 V) (m^2^g^−1^) Cycle 1	MA (0.9 V)/(Ag^−1^) Cycle 6,000	SA (0.9 V)/(µA cm^−2^) Cycle 1	SA (0.9 V)/(µA cm^−2^) Cycle 6,000	Δ MA/%	Δ SA/%
Pt/C	203 ± 7	98 ± 13	350 ± 10	210 ± 13	51.7	40.1
Pt/HCSs	246 ± 17	138 ± 21	387 ± 8	300 ± 11	43.9	22.5
Pt/NHCSs	263 ± 13	139 ± 16	391 ± 16	310 ± 21	47.1	20.7

The area-specific activities (SA) of the catalysts were obtained from the normalization of the kinetic current with the ECSAs of the catalysts. The Pt/NHCSs had a SA of 391 ± 16 A g^−1^, the Pt/HCSs a value of 387 ± 8 A g^−1^, both of which are ca. 10% larger than the commercial Pt/C (350 ± 10 A g^−1^) catalyst (Table 1). The kinetic current of the catalysts was then normalized against the Pt loading on the WE to yield the mass-specific activities (MA) (Figure 6D). The observed trend shows that the Pt/NHCSs (263 ± 13 µA cm^−2^) > Pt/HCSs (246 ± 17 µA cm^−2^) >Pt/C (203 ± 7 µA cm^−2^).

The variation of catalysts activity was attributed to the physicochemical properties of the supports and N doping which affected the electronic properties of the catalysts. The interaction of the Pt and the N groups altered the electronic properties of the Pt-support resulting in better activity and a better Pt-support interaction. From the Pt surface, electron density is transferred to the π-conjugated system on the surface of the nitrogen groups due to differences in electronegativity creating a sea of electron density between the Pt particles and the N-functionalized surface. The same is less true on a pristine carbon surface. Here charge transfer occurs but the higher electronegativity of oxygen terminal functional groups means more of the electron density from the Pt is transferred to the oxygen functional groups. Thus, less of the charge is transferred to the carbon surface. As a result, the sea of charge on the pristine support is smaller than that on the N doped surface. Thus, electron flow is much easier on the N-doped surface than on the pristine surface, resulting in better activity in the former (Panchenko et al., 2004; Ju et al., 2015; Lu et al., 2020).

The high BET surface area of the Pt/HCSs (555 m^2^g^−1^) and Pt/NHCSs (309 m^2^g^−1^) and an array of micro and mesopores promotes better mass transport of oxygen to the Pt active sites compared to the less porous Pt/C benchmark catalyst with a surface area <200 m^2^g^−1^. The larger pores in the Pt/HCSs (5.1 nm, 0.70 cm^3^g^−1^) and Pt/NHCSs (4.1 nm, 0.62 cm^3^g^−1^) facilitates better “in-pore” confinement of the Pt inside the pores and thereby promotes a better Pt-support contact (Supplementary Figure SI4). This is advantageous as it can result in a better support-Pt electronic conductivity and tethering.

### Catalyst Durability

**FIGURE 7:**
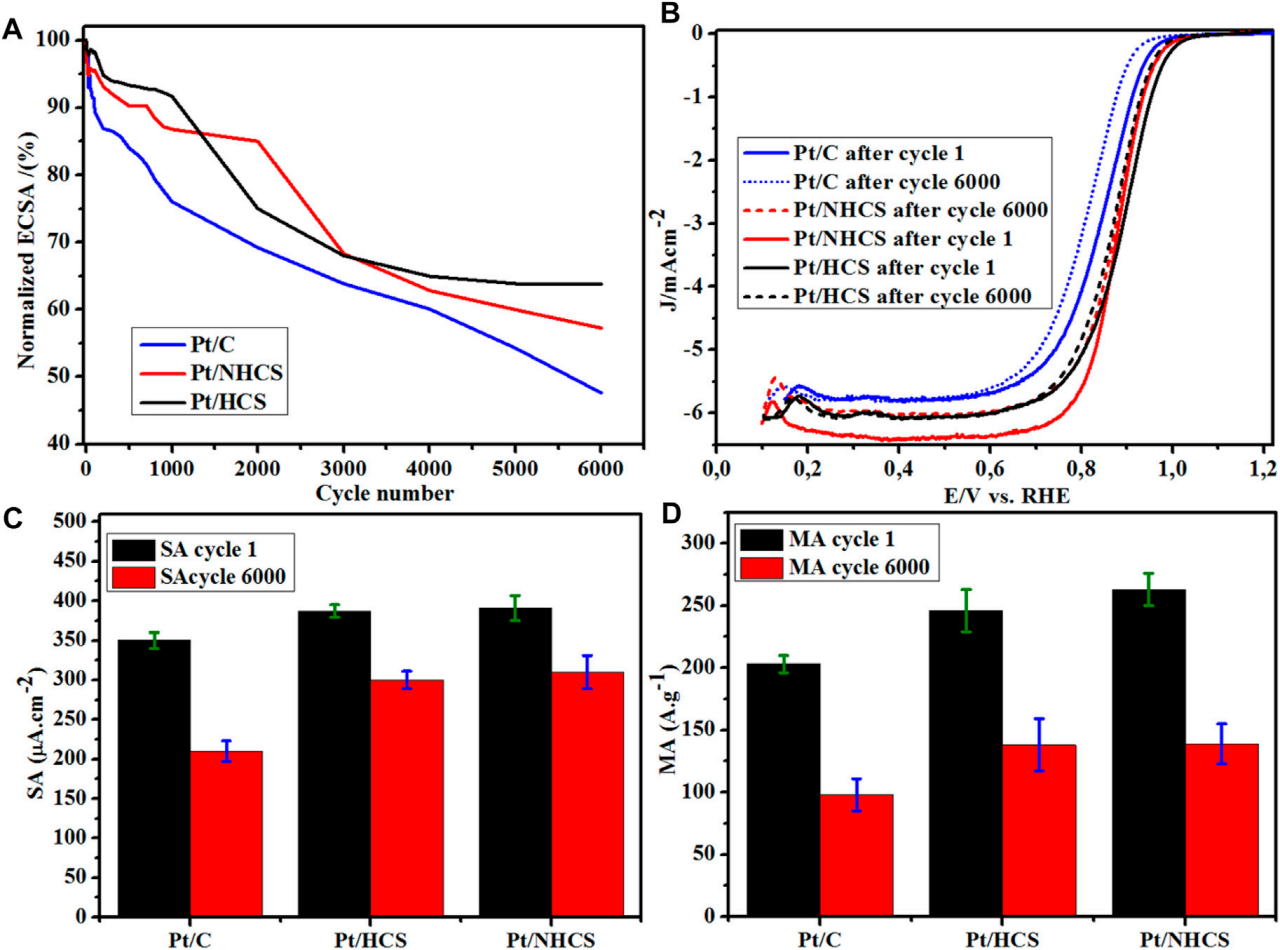
**(A)** Normalized ECSA% degradation of the catalysts, **(B)** catalyst LSVs before and after durability studies for the Pt/HCSs, Pt/NHCSs and the commercial benchmark Pt/C catalysts, **(C)** SA data before and after durability studies, **(D)** MA data before and after durability studies.

The catalysts were subjected to accelerated stress tests (AST) for 6,000 cycles at 50 mV s^−1^ from 0.6–1.0 V vs. RHE (Supplementary Figure SI9). The degradation of the Pt catalysts was observed as a decline in the normalized ECSA % as load cycling was proceeding (Figure 7A). Also observed was the reduction in the size of the regions of the CVs associated with hydrogen adsorption/desorption (Supplementary Figure SI9). The initial 1,000 cycles, saw the benchmark Pt/C maintaining 76.1% of the initial ECSA compared to 94.5% (Pt/HCSs) and 99.8% (Pt/NHCSs). After 5,000 cycles, the benchmark catalysts maintain 54.3% of initial ECSA compared to Pt/HCS (66.2%) and Pt/NHCS (62.1%). After 6,000 cycles, the lowest ECSA retention was observed for Pt/C (47.6%) compared to Pt/HCSs (65.4%) and Pt/NHCSs (62.1%). The decline in ECSA of the catalysts is evident from the comparison of the CVs of the catalyst before and after durability cycling (Supplementary Figure SI10).

The start-stop durability LSVs of the catalysts before and after the tests are shown in Figure 7B. Evident is the shift in the overpotential for the ORR reaction as shown in the catalyst LSVs. The bold lines are cycles before, and the dashed lines are data collected after 6,000 cycles of durability testing (Figure 7B). For the Pt/C catalyst, a measured 30 mV shift in the LSV is observed whereas for the Pt/HCSs (18 mV) and Pt/NHCSs (22 mV), the shifts are smaller. This shift in LSVs is attributed to the loss of activity due to the degradation of the Pt catalysts. The Pt catalysts degrade by dissolution according to the equations

$$\text{Pt}\to {\text{Pt}}^{2+}{+\;2\text{e}}^{-}\;{\text{E}}^{\theta }\;=\;1\text{.}18\text{ V vs SHE}$$ (5)

Or

$${\text{Pt }+\text{ H}}_{2}\text{O }\to {\text{ PtO }+\;2\text{H}}^{+}{\;+\;2\text{e}}^{-}\;{\text{E}}^{\theta }\;=\text{ 0}\text{.}98\text{ V vs SHE}$$ (6)

$${\text{PtO }+\;2\text{H}}^{+}\to {\text{Pt}}^{2+}{+\text{ H}}_{2}\text{O}$$ (7)

The presence of a facial layer of oxide on the surface due to the presence of terminal oxygen functional groups as shown in the O1s spectra of the HCSs is responsible for part of the degradation of the Pt. The formation of the Pt –O bonds weakens the pre-existing Pt–Pt bonds. As the PtO can be further oxidised, higher-order Pt oxidation states such as Pt^4+^ in PtO_2_, undergo dissolution faster than lower-order Pt species according to the equation.

$${\text{PtO}}_{2}{+\;4\text{H}}^{+}{+\;2\text{e}}^{-}\to {\text{ Pt}}^{2+}{+\;2\text{H}}_{2}\text{O}$$ (8)

$${\text{E}}^{\theta }=\text{ 0}\text{.}837\;+\text{ 0}\text{.}0295\text{log }\left[{\text{Pt}}^{2+}\right]$$ (9)

(Cherevko et al., 2016).

The oxidation state of the Pt particles is closely related to Pt dissolution and Ostwald ripening processes. The cathodic dissolution of the Pt particles is a thermodynamically feasible process that occurs below 0.837 V according to Eq. 9. The low dissolution of the Pt and the retention of higher ECSA % on the HCSs and NHCSs based catalysts compared to the commercial catalysts could be attributed to the low oxygen functionalization and subsequently, the existence of lower percentages of higher-order Pt oxidation states. As shown by XPS data, the Pt in the Pt/HCSs and Pt/NHCSs exists mostly as metallic Pt with intact Pt –Pt bonds difficult to dissolve. The observed degradation could be initiated from Pt–O where dissolution led to the formation of Pt^2+^and subsequently Pt^4+^ as further Pt oxidation occurred. Notwithstanding the quantifiable oxygen functionalities on the HCSs and NHCSs supports, significant confinement of the Pt particles inside the pores reduced the rate of migration of dissolved and undissolved Pt particles to form larger Pt clusters.

The shift in the LSVs is also observed in the changes in the MA and SA of the catalysts after durability tests (Figures 7C,D). There is an observed 51.7% change in MA for the benchmark Pt/C catalyst compared to 43.9% (Pt/HCSs) and 20.7% (Pt/NHCSs).

The decline in MA was also coupled with a decline in SA. For the benchmark Pt/C, a 40% decline was observed compared to a 22.4% (Pt/HCSs) and 20.7% (Pt/NHCSs) decline for the two new catalysts. Degradation in fuel cells is known to occur *via* processes that include Pt dissolution, Ostwald ripening, agglomeration, particle detachment as well as support corrosion (Simonsen et al., 2011). These processes result in the formation of bigger particle sizes with less ECSA compared to the initial smaller particles. The smaller ECSA of the newly formed bigger Pt crystallites produces a smaller under potential desorption charge of the hydrogen (Q_DES_), and as shown by Eqs 2, 3 before, there affects the magnitude of the resultant MA and SA values.

The losses in ECSA, MA and SA were attributed to the agglomeration of Pt resulting in the formation of particles that are bigger and with less surface area. The growth in Pt particles can be seen from the comparison of the TEM images before and after stress testing (Supplementary Figure SI11). As shown in these figures, the Pt particle size increased for Pt/HCSs (3.9 ± 0.5 to 4.8 ± 1.2 nm), Pt/NHCSs (3.8 ± 0.6 to 4.9 ± 1.7 nm) and Pt/C (3.9 ± 1.1 to 6.3 ± 1.8 nm).

### Support Durability

The support durability under load cycling conditions was evaluated by subjecting the WE modified by the materials to an accelerated stress test (AST) for 6,000 cycles at 50 mV s^−1^ from 1.0–1.6 V vs. RHE (Supplementary Figure SI12). Under load conditions, the carbon support is known to electrochemically corrode at potentials in the region of 1.5 V vs. RHE according to the equations

$${\text{C }+\;2\text{H}}_{2}\text{O }\to {\text{ CO}}_{2}{+\;4\text{H }+\;4\text{ e}}^{-}\mathrm{tt}\text{​}\text{​}\text{​}\;\;\;{\text{E}}^{\theta }=\text{ 0}\text{.}2\text{ V vs SHE}$$ (10)

Even though the thermodynamic potential for this process is lower (Eq. 10), at higher cell potentials of about 1.5 V vs. RHE, experimental data has shown that this oxidation process is significant (Labata et al., 2021).

**FIGURE 8:**
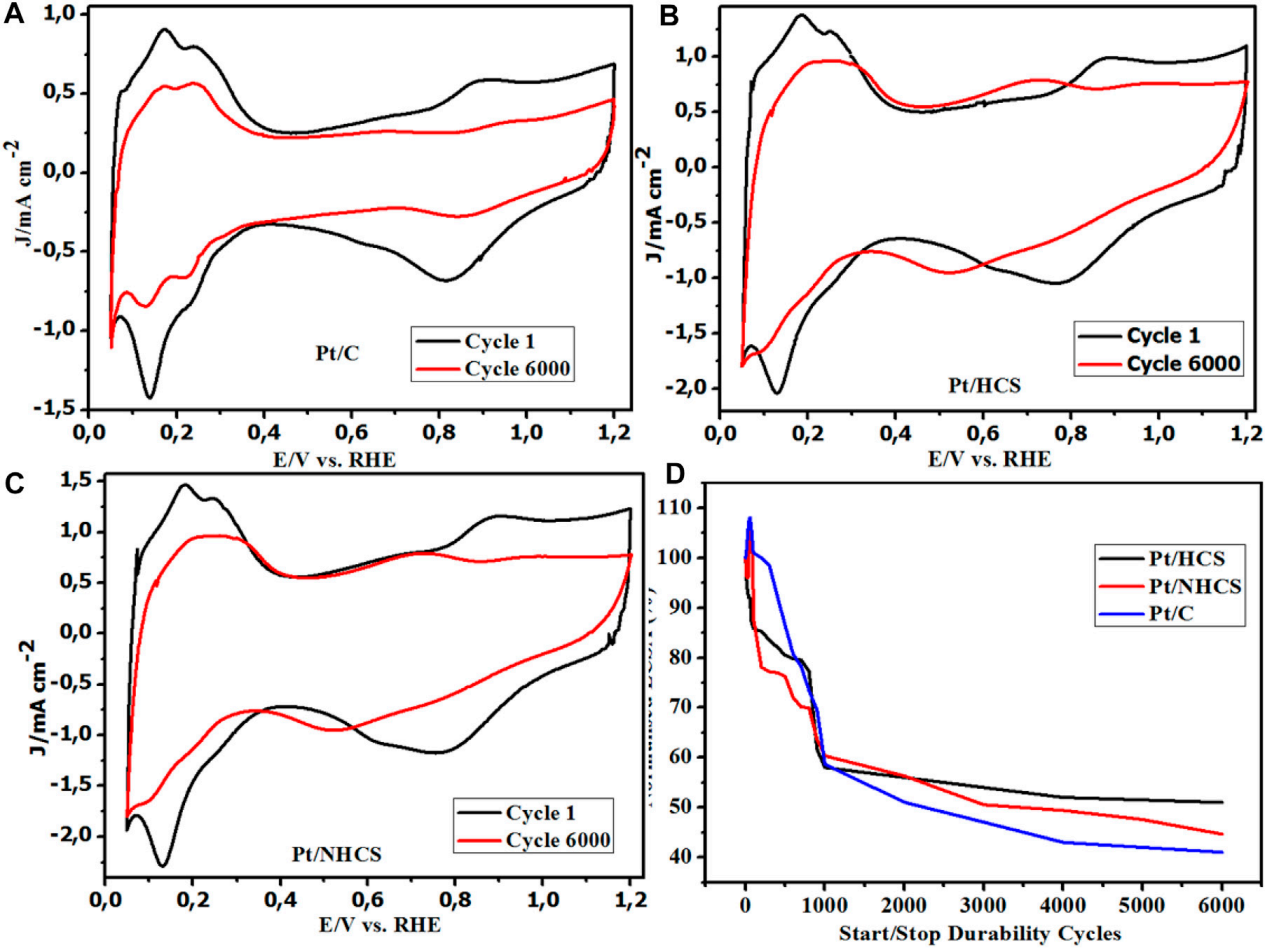
**(A–D)** Catalyst CVs before and after durability studies for the Pt/HCSs, Pt/NHCSs and the commercial benchmark Pt/C catalysts, **(D)** Normalized ECSA% degradation of the catalysts.

The load cycling durability CVs shows a comparison between the before and after AST tests (Figures 8A–C). The CVs show the formation of the quinone/hydroquinone (Q-HQ) couple at about 0.60 V vs. RHE for the catalysts. This redox couple appears as a result of the reversible oxidation of the carbon support according to the equation

$$\text{C}=\text{O}\left(\text{s}\right){\;+\text{ H}}^{+}{+\text{ e}}^{-}\to \text{ C}-\text{OH}$$ (11)

The Q-HQ is a reversible process that does not represent the mass loss of carbon leading to the formation of CO_2_ (Park et al., 2014). However, it demonstrates the susceptibility to oxidation of the carbon and its propensity for final corrosion.

There is a significant formation of the quinone/hydroquinone couple in the Pt/HCSs and the Pt/NHCSs compared to the commercial Pt/C. This could be due to the high surface area and a high degree of functionalization with O (and N groups) as confirmed in the XPS C1s and O1s spectra. This presence results in the ease of interfacial oxidation of the thin layers of carbon. The low formation of the Q-HQ couple on the commercial benchmark could be attributed to the low BET surface area as a result of the carbon degradation to CO_2_ as well as the presence of fewer graphite-like edge sites at which the hydroxyl functional groups could form during the corrosion process according to Eq. 11. The reduction is attributed to the loss of the catalysts ECSA due to the degradation of the carbon supports resulting in migration of the Pt particles and subsequent agglomeration. The other observable feature is the growth of the double-layer capacitance of the Pt/HCSs and Pt/NHCSs catalysts in the 0.3–0.5 V vs. RHE potential window corresponding to the reduced surface area of the catalysts.

Even though the double-layer capacitance and the features associated with the ECSA of the catalysts are reduced, due to degradation of the Pt/HCSs and the Pt/NHCSs catalysts, more degradation is observed for the commercial Pt/C catalyst (Figure 8). The AST study resulted in a 59%, 57% and about 61% reduction of the initial ECSA for the Pt/NHCSs, Pt/HCSs and Pt/C catalysts respectively after 6,000 cycles (27 h). The change in ECSA from the 1,000 durability cycles to the 6,000 durability cycles shows a minimal reduction occurring for the Pt/HCSs accounting for a 6% loss. Significant losses are observed for the Pt/C (18%) and the Pt/NHCSs (15%).

The slightly better durability of the HCSs-based supports is attributed to the structure of these materials. The HCSs (832 m^2^/g) and NHCSs (604 m^2^/g) supports both have a higher BET surface area and an array of mesopores and micropores which promotes the confinement of Pt particles, resulting in better support to metal contact. Also, confined Pt particles are more resistant to migration and agglomeration. The higher loss in activity of the benchmark Pt/C is ascribed to low pore confinement compared to the new materials. Pore confinement of the Pt in the structure of the HCSs based supports was confirmed in a similar study involving broken hemispherical hollow carbon spheres (Mashindi et al., 2021).

Overall, the confinement of the Pt inside the pores as shown by ABF-DF-STEM (Supplementary Figure SI4) and related work with similar materials (Mashindi et al., 2021), as well as the good Pt-support contact due to surface functionalization is critical for better durability under aggressive fuel cell cycles. Equally important is the elongation of bonds and the presence of higher-order rings in the structure of the carbon that results in a poor carbon-carbon bond strength, which produces a reduced stability in the nitrogen-doped support relative to the pristine hollow carbon spheres as elucidated by the PDF analysis of the samples.

## Conclusion

In this study, the various properties of the HCSs and NHCSs catalysts were studied, and correlated to the observed durability and activity in ORR. Characterization studies on the HCSs, NHCSs and Pt/HCSs and Pt/NHCSs catalysts revealed that the porosity of the supports and their high surface areas promoted good dispersion of the Pt nanoparticles as revealed by BET analysis. Raman spectra indicated that the nitrogen and oxygen functional groups generated numerous defects on the surface of the support where nucleation and growth of small Pt particles was initiated.

The effect of nitrogen on the HCSs structure from total scattering data using pair distribution function (PDF) analysis of the catalysts was studied. Analysis of the data revealed that the NHCSs showed increased buckling of the carbon plane compared to the pristine sample due to the nitrogen doping. Addition of Pt to the spheres also gave rise to a Pt-C interaction that could be detected by PDF analysis and the strength of the interaction of the Pt particles with the carbon surface is such that the larger the interaction the larger the C–C bond elongation. The stronger Pt-C interaction was responsible for part of the observed long-term durability of the Pt/HCSs and Pt/NHCSs catalysts.

The PDF method is highly sensitive to small particles resulting in an accurate particle size determination. The higher proportion of smaller average Pt sizes as determined by the PDF technique compared to PXRD, could partly explain the higher activity in ORR observed for the Pt/HCSs and Pt/NHCSs.

The Pt catalysts prepared by the ethanol reflux method demonstrated higher activity and durability than the commercial benchmark Pt. The durability the Pt catalysts were subjected to accelerated stress tests (AST) for 6,000 cycles at 50 mV s^−1^ from 0.6 to 1.0 V vs. RHE. The data showed that after 6,000 cycles, the ECSA retentions were Pt/C (47.6%) < Pt/NHCSs (62.1%) < Pt/HCSs (65.4%). In other experiments the start-stop durability LSVs of the catalysts, before and after the tests, was studied and the data revealed LSV shifts in the order Pt/HCSs (18 mV) < Pt/NHCSs (22 mV) < Pt/C (30 mV). A shift in LSVs is attributed to the loss of activity due to the degradation of the Pt catalysts. The losses in ECSA, MA (and SA) were attributed to the agglomeration of Pt and this agglomeration was detected by post analysis of the samples from TEM images.

The two effects of pore confinement and the presence of defects/nucleation sites is proposed to be responsible for the enhanced durability of the catalysts. Based on these findings, the Pt/HCSs and Pt/NHCSs are good candidates for PEMFC catalysts and strategies to further enhance the durability of the catalysts and reduce Pt use can be further initiated from the above studies.

## Data Availability

The original contributions presented in the study are included in the article/Supplementary Material, and further inquiries can be directed to the corresponding author.
