# Supplementary material for: Platinum Nanocatalysts Supported on Defective Hollow Carbon Spheres: Oxygen Reduction Reaction Durability Studies
Source: Front Chem. 2022 Feb 21;10:839867. doi: 10.3389/fchem.2022.839867 (PMC8899172; doi:10.3389/fchem.2022.839867)
Supplement: Supplementary file 1 [file DataSheet1.docx]

**Platinum nanocatalysts supported on defective hollow carbon spheres: oxygen reduction reaction durability studies**

Victor Mashindi^1^, Pumza Mente^1^, Tumelo Pahlamohlaka^1^, Nobuhle Mpofu^2^, Ofentse Makgae^3^, Beatriz D. Moreno^4^, Dean H. Barrett^1^, Roy Forbes^1^, Kenneth I. Ozoemena^1^, Pieter B. Levecque^2^ and Neil J. Coville^1^*,

^1^Molecular Sciences Institute, School of Chemistry, University of the Witwatersrand, Johannesburg 2050, South Africa

^2^ HySA Catalysis Centre of Competence, Catalysis Institute, Department of Chemical Engineering, University of Cape Town, 7701, South Africa

^3^ National centre for high resolution electron-microscopy (nCHREM), Centre for Analysis and synthesis NanoLund, Lund University, Naturvetarvagen 14 PO Box 124, 221 00 Lund, Sweden

^4^Canadian Light Source Inc., 44 Innovation Boulevard, Saskatoon, SK S7N 2V3, Canada

*Prof Neil Coville. E-mail: neil.coville@wits.ac.za. Tel: +27 (0) 11 717 6738.


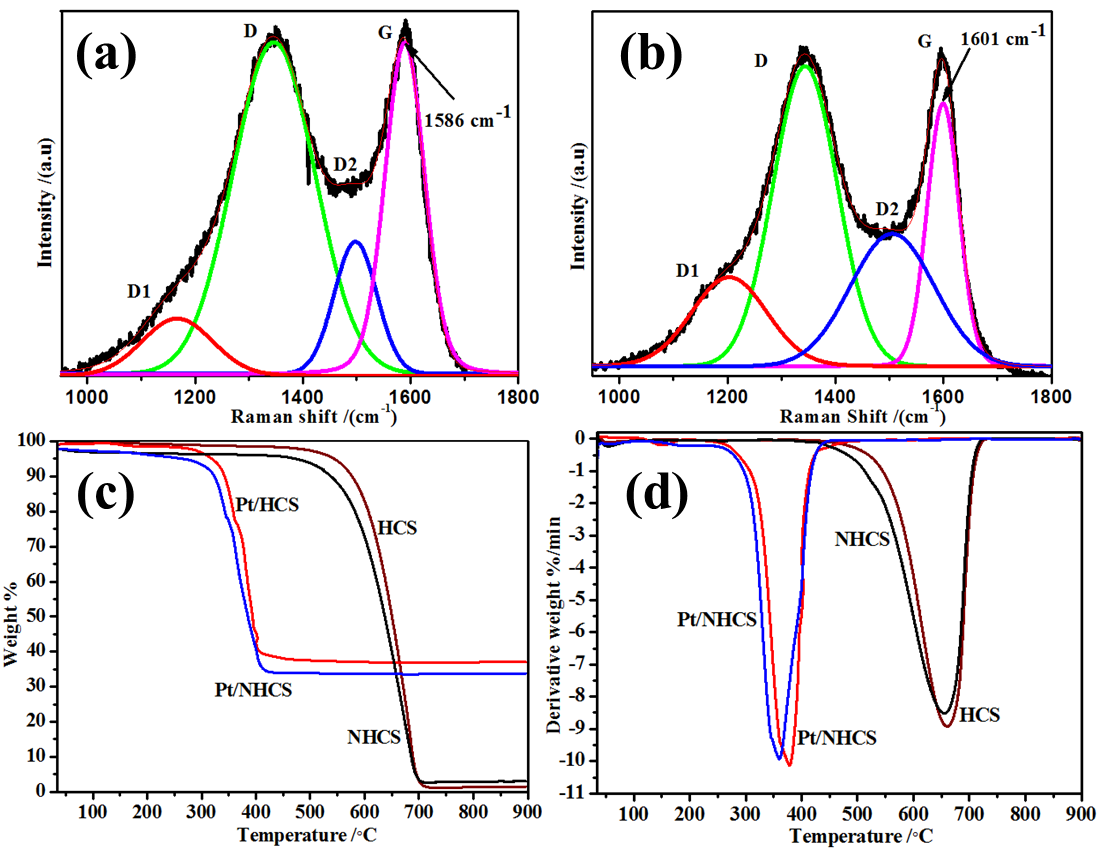


Figure SI 1: Raman spectra of HCSs (a) Raman spectra of NHCSs (b) TGA profiles of HCSs, NHCSs, Pt/HCSs and Pt/NHCSs (c) TGA derivative profiles of HCSs, NHCSs, Pt/HCSs and Pt/NHCSs


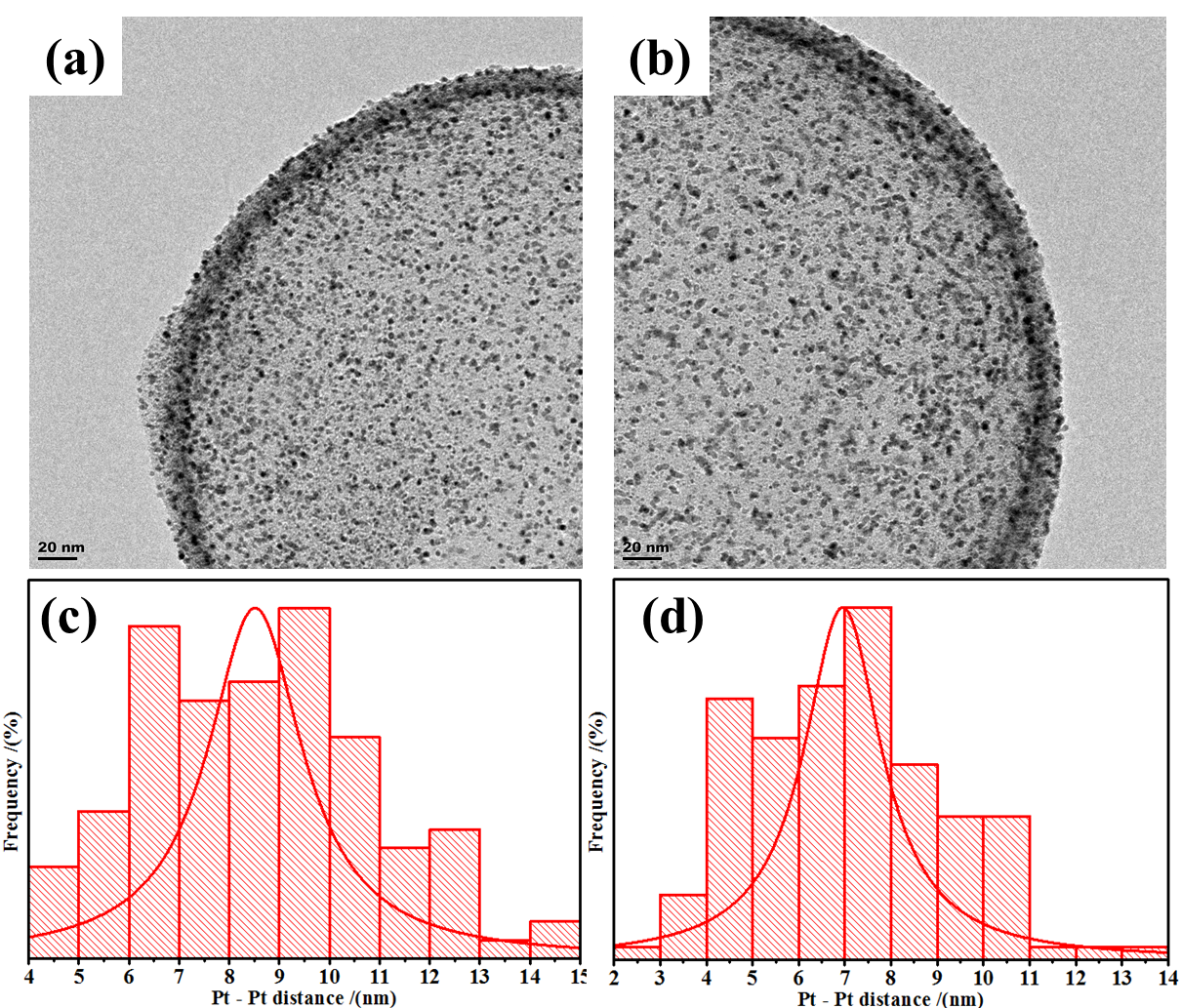


Figure SI 2: TEM image of (a) Pt/HCSs (b) Pt/NHCSs (c) Pt – Pt interparticle distance of Pt/HCSs (d) Pt – Pt interparticle distance of Pt/NHCSs.


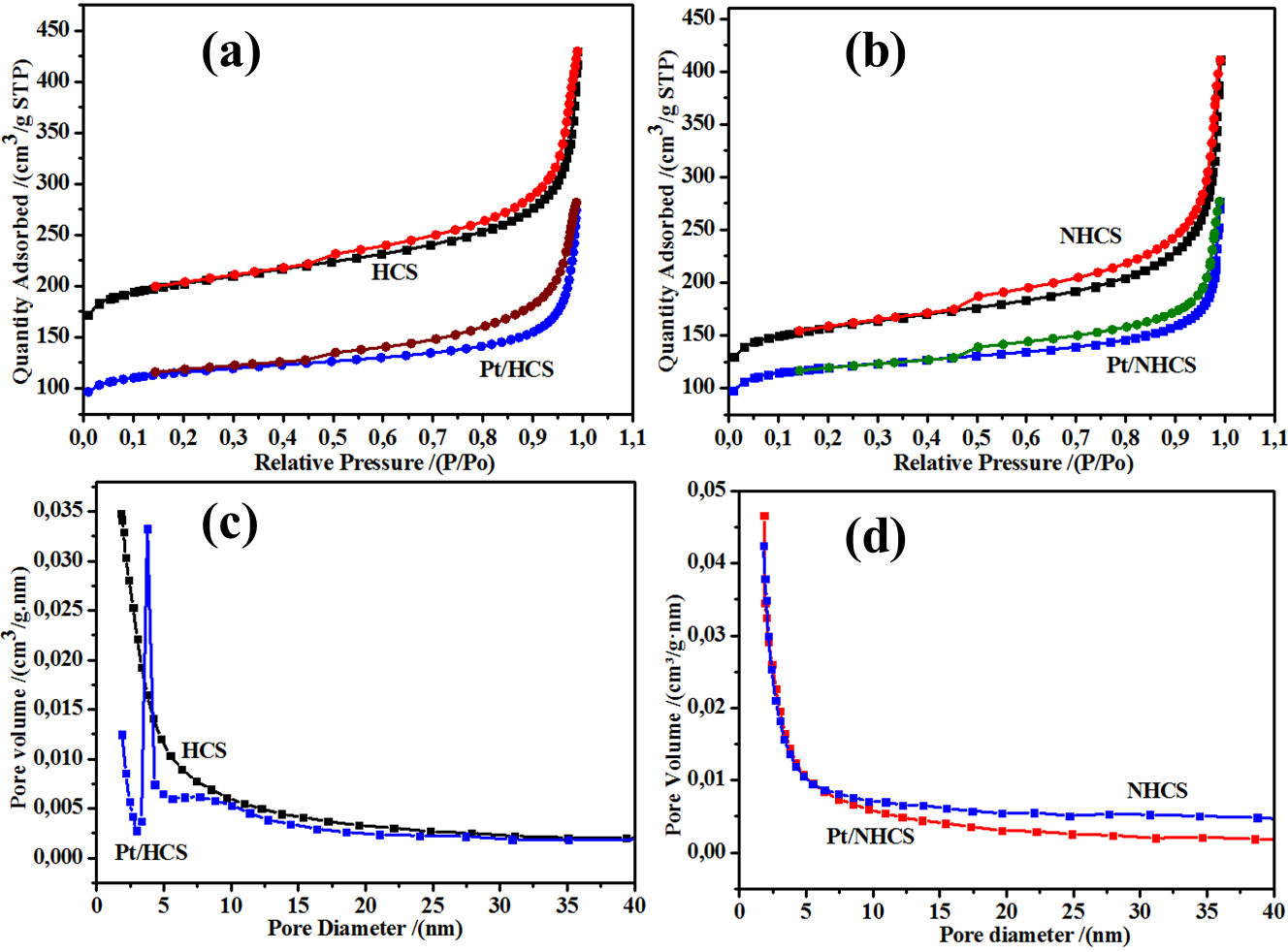


Figure SI 3: BET isotherms for (a) HCSs and Pt/HCSs, (b) NHCSs and Pt/NHCSs. Pore size distribution plots for (c) HCSs and Pt/HCSs and (d) NHCSs and Pt/NHCSs.

Table SI 1: Textual properties of HCSs, NHCSs, Pt/HCSs and Pt/NHCSs

| Sample | BET SA m^2^/g | Micropore SA m^2^/g | % micropore  SA | Pore Volume cm^3^/g | Micropore volume cm^3^/g | % micropore  volume | Pore Width /nm |
| --- | --- | --- | --- | --- | --- | --- | --- |
| HCS | 832 | 595 | 72 | 0.53 | 0.29 | 55 | 12.1 |
| Pt/HCS | 555 | 343 | 62 | 0.70 | 0.18 | 26 | 5.1 |
| Δ | 277 | 252 | 91 | 0.22 | 0.10 | 45 | 7 |
| NHCS | 604 | 322 | 53 | 0.62 | 0.16 | 26 | 8.3 |
| Pt/NHCS | 309 | 199 | 64 | 0.35 | 0.10 | 29 | 4.1 |
| Δ | 295 | 123 | 42 | 0.27 | 0.06 | 22 | 4.2 |


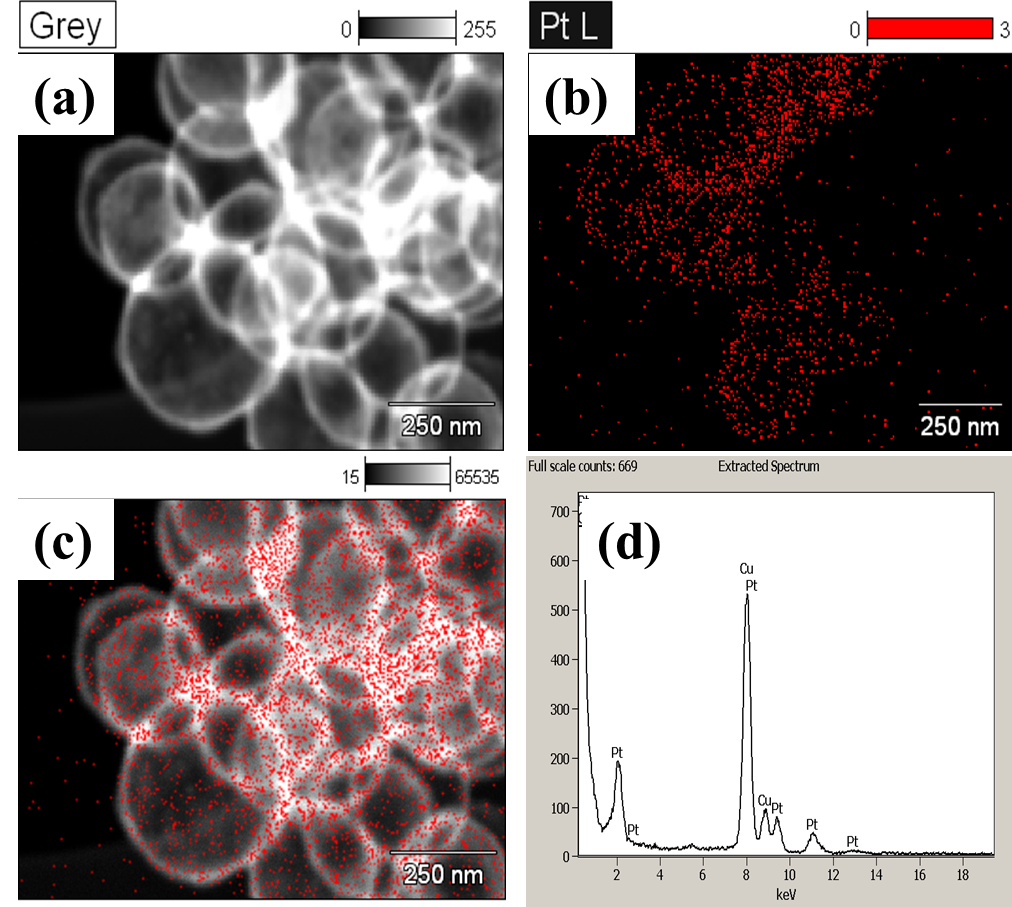


Figure SI 4: (a) DF-STEM images of Pt/HCSs showing the bright edges of the HCSs support due to Pt. (b) and (c) Pt elemental map showing location of Pt in the shell region of the support. (d) EDX showing the presence of Pt on the supports, the copper is due to the copper grid used for analysis.


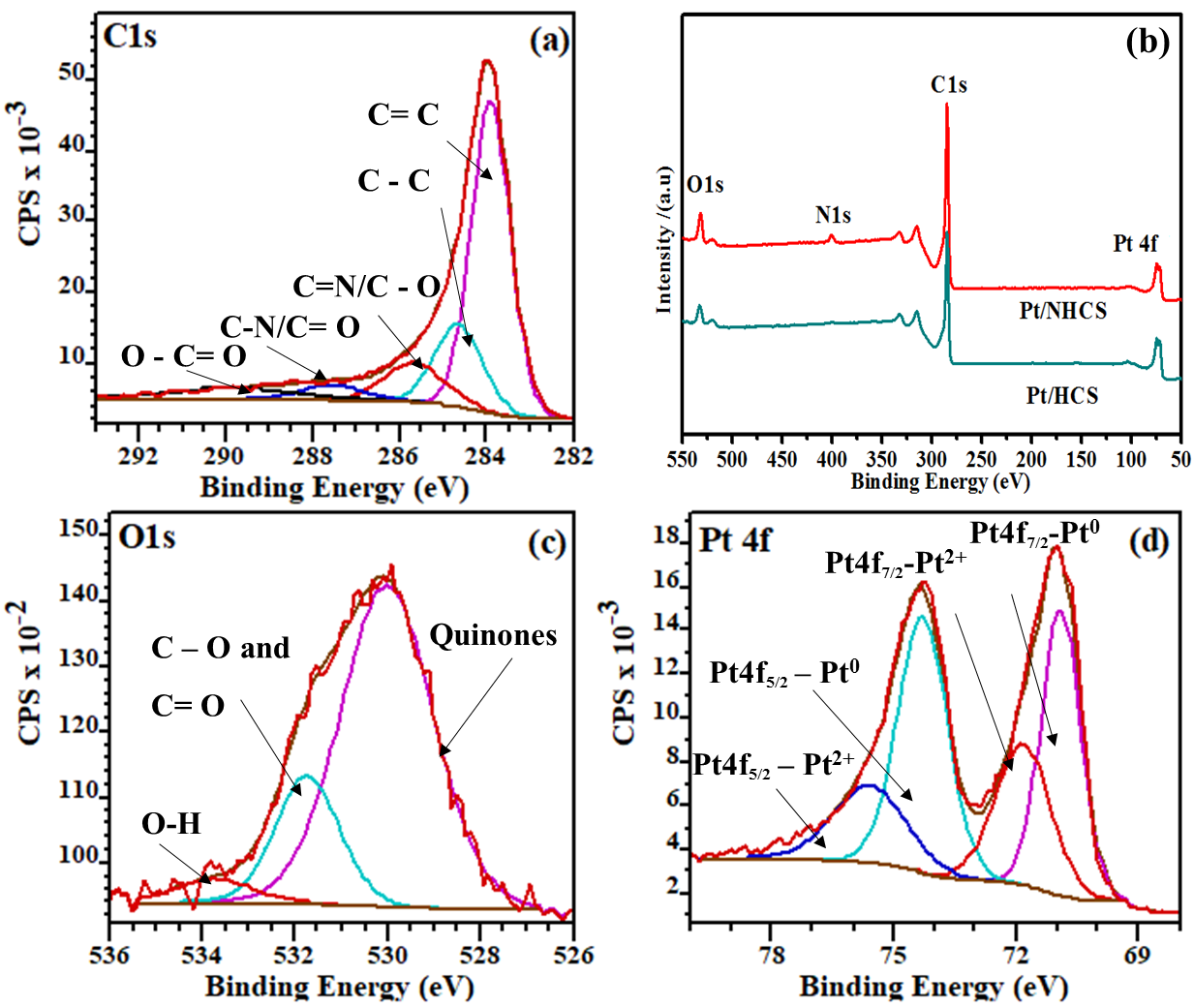


Figure SI 5: Deconvoluted high-resolution XPS spectra for Pt/HCSs (a) C1s, (b) survey spectra for Pt/HCSs and Pt/NHCSs (c) deconvoluted high-resolution XPS spectra for Pt/HCSs O1s and (d) Pt 4f.


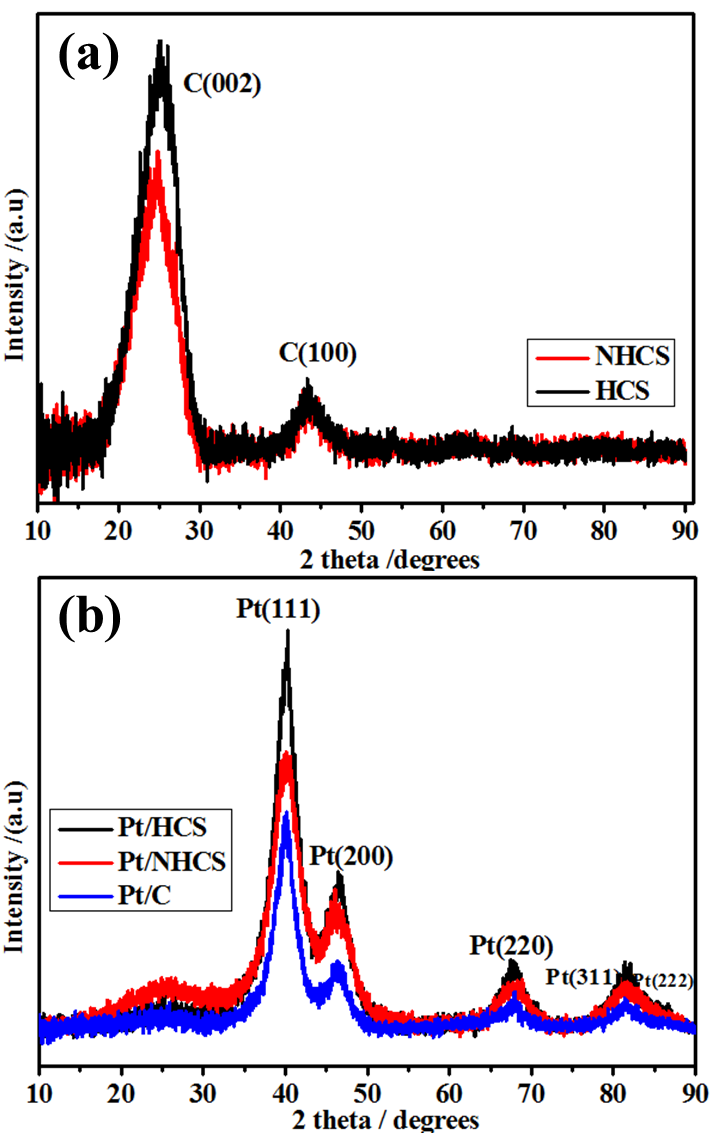


Figure SI 6: PXRD diffractograms of (a) HCSs and NHCSs, (b) Pt/HCSs and Pt/NHCSs


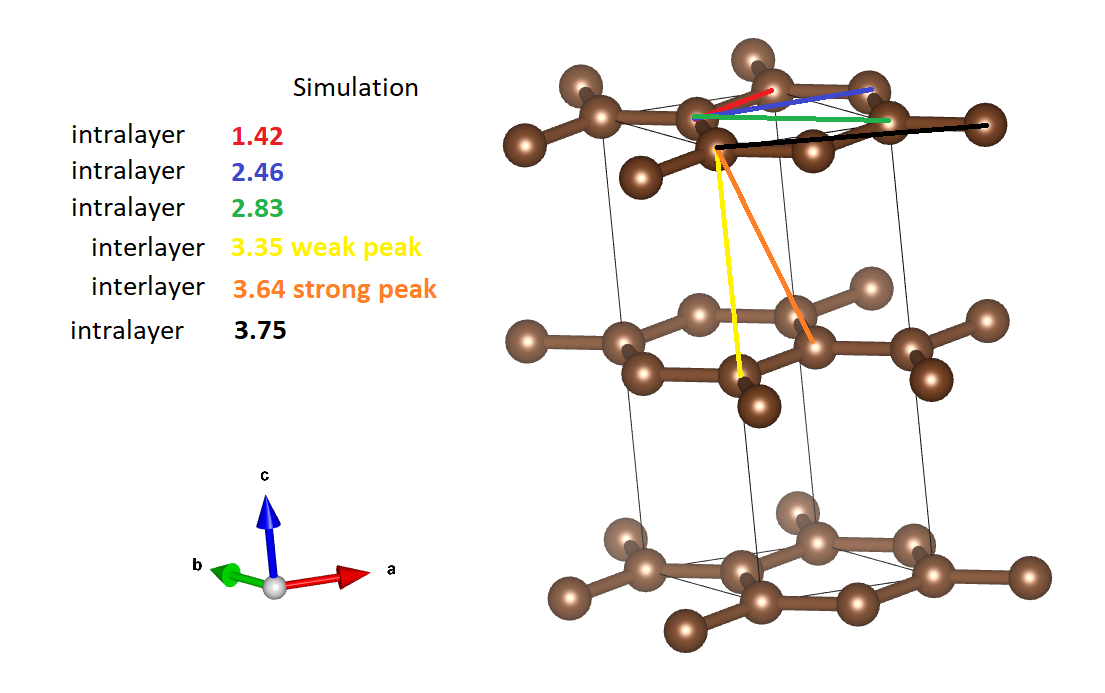


Figure SI 7: Graphite C-C bond distances


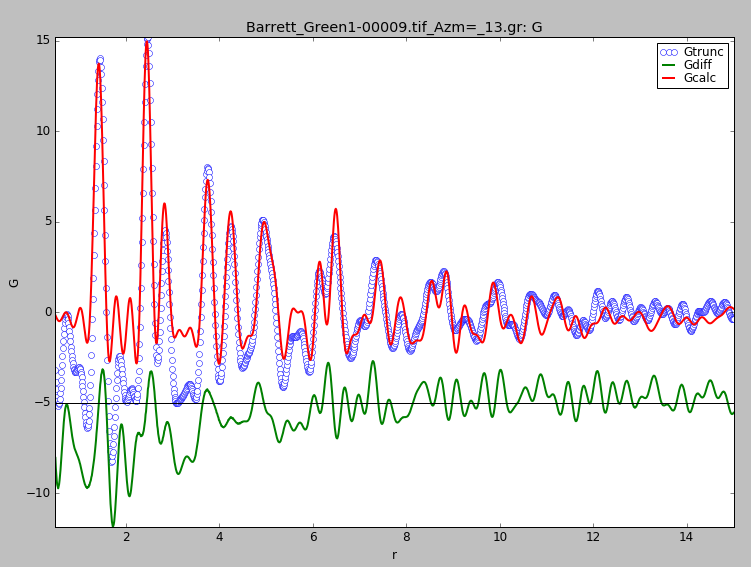


Figure SI 8. PDF fitting of graphite structure to measured data.


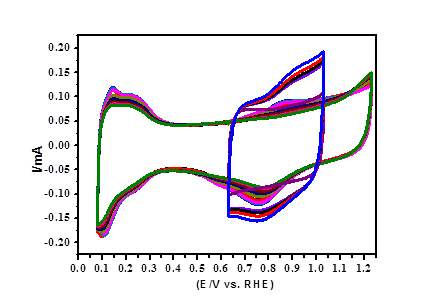


Figure SI 9: Cyclic voltammograms for ECSA and load cycling (catalyst durability cycle) recorded in 0.1 M HClO_4_ (room temperature and in argon saturated electrolyte) for Pt/HCSs


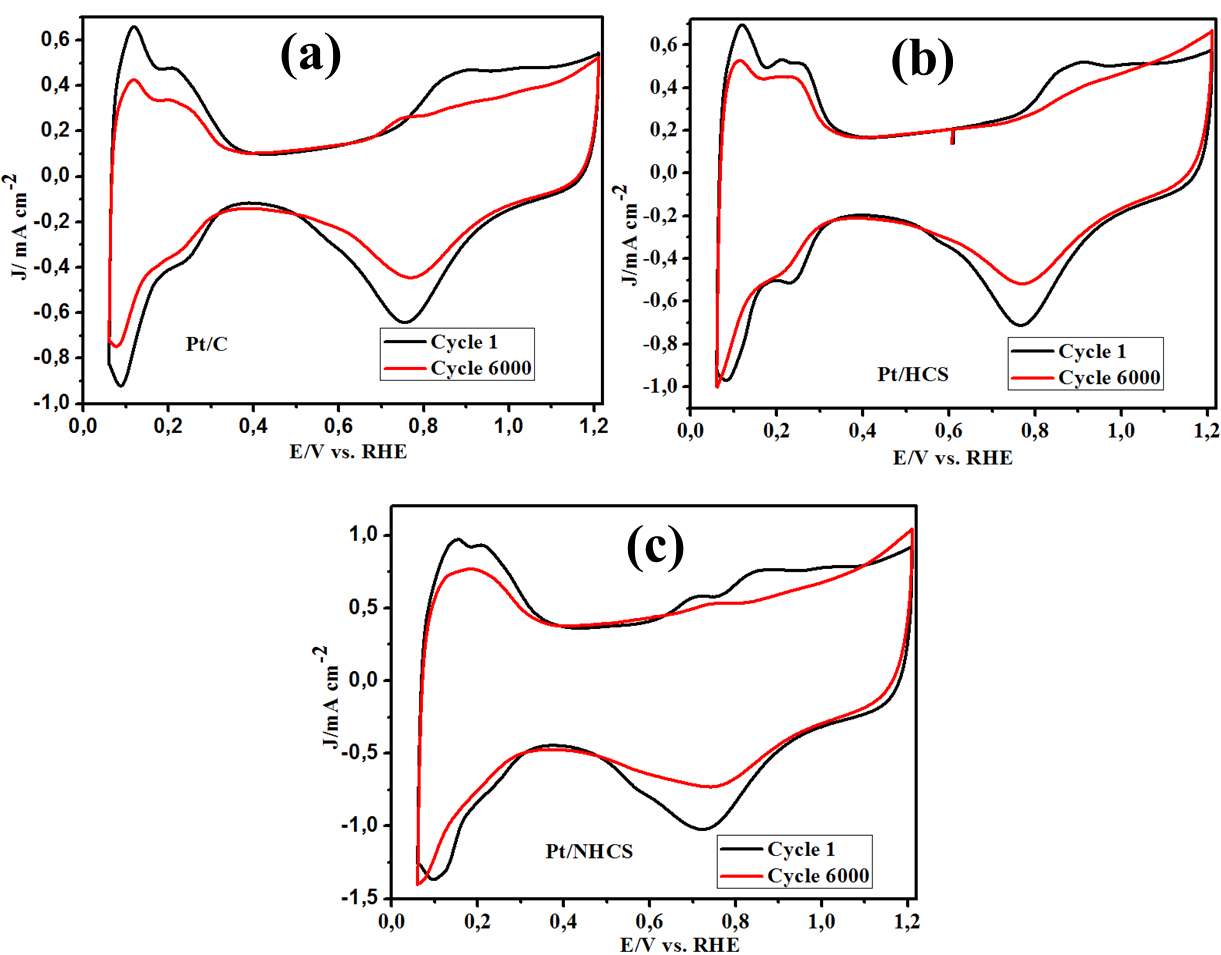


Figure SI 10: Catalyst CVs before and after durability studies for the (a) Pt/C, (b) Pt/HCSs and (c) Pt/NHCSs


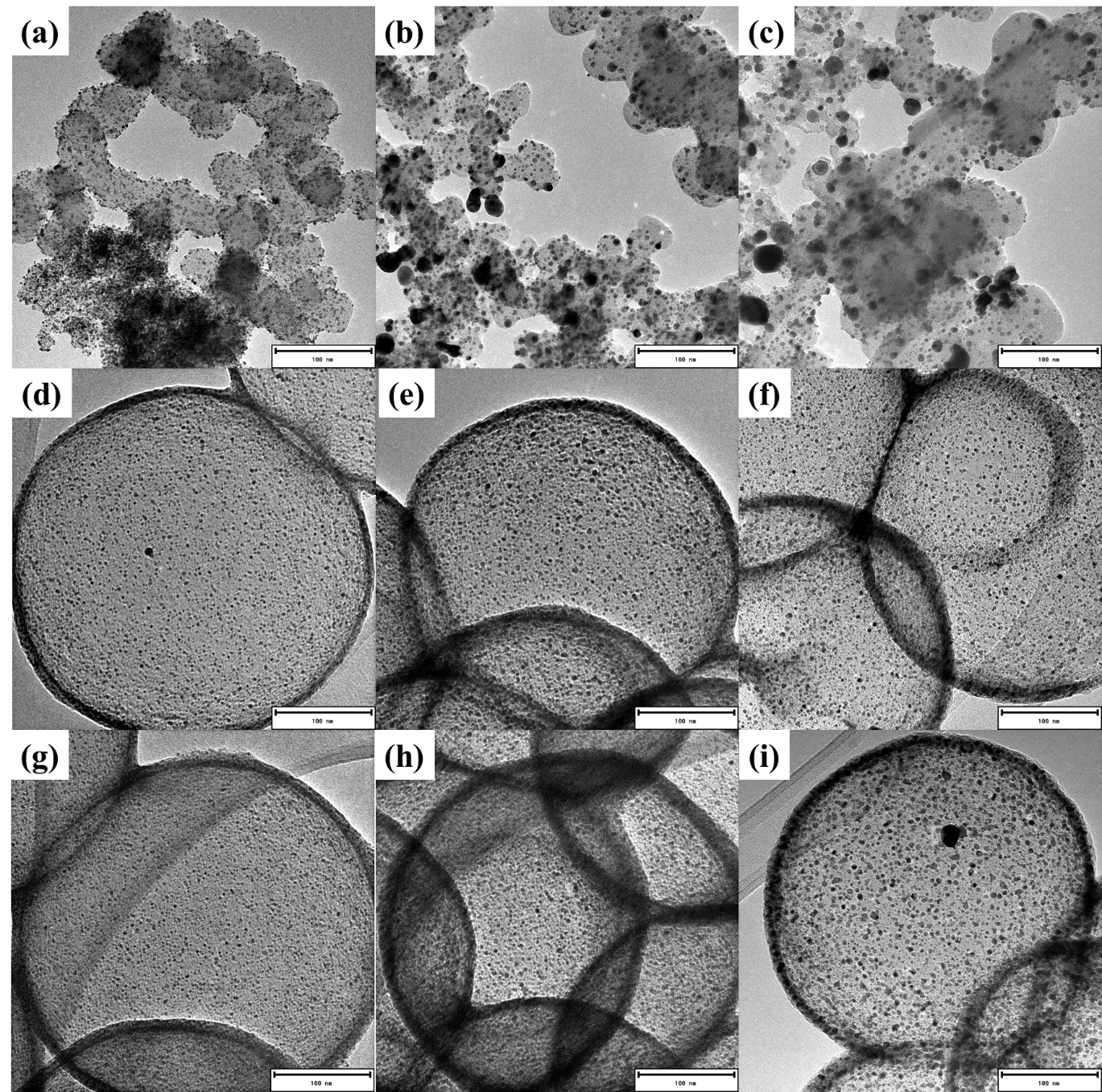


Figure SI 11: After durability study TEM image of (a) Pt/HCSs (b) Pt/NHCSs (c) particle size distribution of Pt on Pt/HCSs (d) particle size distribution of Pt on Pt/NHCSs.


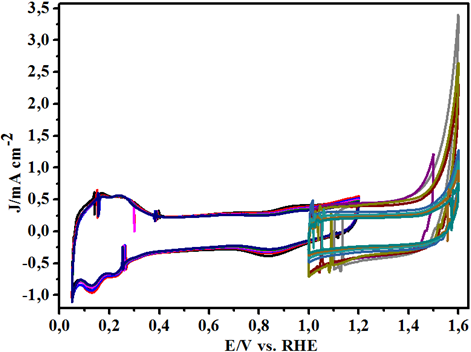


Figure SI 12: Cyclic voltammograms for ECSA and load cycling (support durability cycle) recorded in 0.1 M HClO_4_ (room temperature and in argon saturated electrolyte) for Pt/C.

.
